# TrAGEDy—trajectory alignment of gene expression dynamics

**DOI:** 10.1093/bioinformatics/btaf073

**Published:** 2025-03-11

**Authors:** Ross F Laidlaw, Emma M Briggs, Keith R Matthews, Amir Madany Mamlouk, Richard McCulloch, Thomas D Otto

**Affiliations:** Centre for Parasitology, University of Glasgow, Glasgow, G12 8QQ, United Kingdom; Centre for Parasitology, University of Glasgow, Glasgow, G12 8QQ, United Kingdom; Institute for Immunology and Infection Research, University of Edinburgh, Edinburgh, EH8 9YL, United Kingdom; Biosciences Institute, Newcastle University, Newcastle upon Tyne, NE1 7RU, United Kingdom; Institute for Immunology and Infection Research, University of Edinburgh, Edinburgh, EH8 9YL, United Kingdom; Institute for Neuro- and Bioinformatics, University of Lübeck, Lübeck, 23562, Germany; Centre for Parasitology, University of Glasgow, Glasgow, G12 8QQ, United Kingdom; Centre for Parasitology, University of Glasgow, Glasgow, G12 8QQ, United Kingdom; Laboratory of Pathogens and Host Immunity, Universite de Montpellier, Montpellier, 34090, France

## Abstract

**Motivation:**

Single-cell transcriptomics sequencing is used to compare different biological processes. However, often, those processes are asymmetric which are difficult to integrate. Current approaches often rely on integrating samples from each condition before either cluster-based comparisons or analysis of an inferred shared trajectory.

**Results:**

We present Trajectory Alignment of Gene Expression Dynamics (TrAGEDy), which allows the alignment of independent trajectories to avoid the need for error–prone integration steps. Across simulated datasets, TrAGEDy returns the correct underlying alignment of the datasets, outperforming current tools which fail to capture the complexity of asymmetric alignments. When applied to real datasets, TrAGEDy captures more biologically relevant genes and processes, which other differential expression methods fail to detect when looking at the developments of T cells and the bloodstream forms of *Trypanosoma brucei* when affected by genetic knockouts.

**Availability and implementation:**

TrAGEDy is freely available at https://github.com/No2Ross/TrAGEDy, and implemented in R.

## 1 Introduction

First described in 2014 (Trapnell *et al.* 2014), trajectory inference (TI) methods order cells based on the gradual change in transcript levels in underlying biological processes captured by single-cell RNA sequencing (scRNA-seq). Cells are assigned a ‘pseudotime’ value based on their position in the inferred trajectory, allowing differential gene expression tests over pseudotime. Various methods have been developed to identify differentially expressed (DE) genes in a biological process of interest, in some cases allowing targeted comparisons. Notably, TradeSeq (Van den Berge *et al.* 2020) uses negative binomial general additive models (GAMs) and Wald tests to assess whether a gene is DE over pseudotime within a trajectory or between lineages of the same trajectory. Alternatively, Monocle’s BEAM (Qiu *et al.* 2017) identifies genes associated with a particular branch of a trajectory, whereas pseudotimeDE (Song and Li 2021) uses permutation to account for uncertainty in pseudotime and a zero-inflated negative binominal GAM to account for expression value dropout. By applying these techniques, dynamic gene expression patterns associated with a variety of interesting biological events have been investigated, such as regulators of myogenesis (Trapnell *et al.* 2014), effector gradients in CD4+ T cells (Cano-Gamez *et al.* 2020), and life cycle transitions of the pathogen *Trypanosoma brucei* (Briggs *et al.* 2021).

Some of these methods can be extended to identify DE genes between two conditions where the common biological process under analysis varies, such as before and after mutation or in an overlapping response to different stimuli (Van den Berge *et al.* 2020). One approach is to integrate the datasets together, perform TI to capture the shared, possibly branching, trajectory, and differential expression tests across this pseudotime axis for each condition, using tools like TradeSeq’s conditionTest. However, the integration may force similarity between the trajectories, thus obscuring DE genes.

Alternatively, trajectory alignment can align cells from independently generated trajectories to find a common pseudotime axis that retains the original ordering of cells without dataset integration. Analysing aligned trajectories can reveal DE genes across the captured process, as well as differences between conditions. The discrete point at which the genes are DE between conditions can also be found. The first method described to perform such trajectory alignment was cellAlign (Alpert *et al.* 2018), which uses dynamic time warping (DTW) to align two trajectories together. The use of DTW imposes some constraints on the type of alignment that can be analysed. Each trajectory being compared must have the same start and end points, and each part of a trajectory must be matched to at least one point in the other trajectory. The practical implication of such limitations is that cell types that may not be represented on the opposing trajectory are nevertheless matched to a point on the other trajectory, making interpretation of the subsequent alignment difficult.

Here, we built on the work of cellAlign with Trajectory Alignment of Gene Expression Dynamics (TrAGEDy). We show that, through our approach to remove the constraints of DTW, TrAGEDy can accurately capture partial alignments of simulated scRNA-seq datasets where current methods fail, highlighting the importance of TrAGEDy in scenarios when comparing an unknown cell type and its position in development to a known developmental progression. We also show that performing DE gene analysis between the trajectories with TrAGEDy allows the identification of more biologically relevant genes than existing methods.

## 2 Materials and methods

The following is an overview of the steps that TrAGEDy takes to analyse a dataset. An outline of the TrAGEDy process can be seen in Supplementary Fig. S1 and implementation of the method can be seen, in full, in the code contained on GitHub (https://github.com/No2Ross/TrAGEDy).

### 2.1 Create interpolated points

As input, TrAGEDy takes two scRNA-seq datasets for which pseudotime values have been calculated (Supplementary Fig. S1A). Performing alignment directly on every cell in a dataset would be computationally and time expensive and prone to noise. As such, a modification of the cellAlign method was used to smooth gene expression over the trajectory by creating a user-defined number of interpolated points that sample the gene expression patterns of surrounding cells at specific points in the process and perform alignment on them. Each interpolated point is given the same, user defined, window of pseudotime around it: *windowSize*. For this article, *windowSize* was set to be the maximum pseudotime divided by 90% of the number of interpolated points. This ensures that *windowSize* is proportional to the number of interpolated points used and allows overlap in the window edges between interpolated points. Cells within the window contribute greatly to the gene expression of the interpolated point, while ones further away contribute less. TrAGEDy weights *windowSize* by the density of cells around each interpolated point. Interpolated points with many cells around it will have a smaller window, while those with few surrounding cells will have a larger window (Supplementary Fig. S1B).

Let $X\in R^{g\times c}$ contain normalized scRNA-seq expression counts, where $X_{k,l}$ is the normalised expression value of gene *k* in cell *l* and *c* is the number of cells. The *g* genes are a subset of the total number of genes captured in the scRNA-seq experiment which aims to define the biological process being aligned. These genes can be highly variable genes, or marker genes of clusters derived from the datasets being aligned. The trajectories aligned with TrAGEDy must have the same *g* genes.

Let $p\in R^{c}$ where *p_l_* is the pseudotime value of cell *l* and let $q\in R^{u}$ where *q_h_* is the pseudotime value of interpolated point *h*. The number of interpolated points, *u*, is specified by the user. For this article, the number of interpolated points was chosen to be in the range of 10–20 interpolated points per cluster in the dataset, with both datasets getting the same number of interpolated points as the dataset with the largest number of clusters. This was to ensure there was no input bias as to which dataset may be longer than the other.

Finally, let $\mathrm{qTotal}\in R^{u}$, where *qTotal_h_* is the number of cells that fall within $q_{h}\;\pm \;\mathrm{windowSize}$ of interpolated point *h*:


$$\mathrm{qTota}l_{h}=\sum_{l\;=\;1}^{c}s_{l}$$

$$\mathrm{where}\;s_{l}=\{\begin{array}{cc} 1 & \left(q_{h}-\mathrm{windowSize})\leq p_{l}\leq (q_{h}+\mathrm{windowSize}\right)\\ 0 & \text{else} \end{array}$$

Let $\mathrm{qWindowSize}\in R^{u}$, where, for each interpolated point, *qWindowSize* defined as:


$$\mathrm{qWindowSiz}e_{h}=\mathrm{windowSize}\times (1-\frac{\mathrm{qTota}l_{h}-\text{mean}(\mathrm{qTotal})}{\text{max}(\mathrm{qTotal})})$$

This vector contains the adjusted window sizes for the interpolated points, with interpolated points with more cells than the mean getting a smaller window size and interpolated points with less cells than the mean getting a larger window size. The adjusted window sizes, normalized gene expression matrix *X* and cell pseudotimes *p* are then fed into the cellAlign (Alpert *et al.* 2018) algorithm for calculating gene expression values for the interpolated points (Supplementary Fig. S1B).

### 2.2 Scoring dissimilarity

Let $O=(O_{i}:i\in \{1,\ldots ,n\})$ and $T=(T_{j}:j\in \{1,\ldots m\}$ denote the two time series being aligned during the TrAGEDy procedure, where *n* is the number of interpolated points in *O* and *m* the number of interpolated points in *T*. For every interpolated point in *O* and *T*, we calculate the transcriptomic dissimilarity between it and all the interpolated points on the other trajectory and store it in a matrix. We define a matrix $D\in R^{n\times m}$, where each element $D_{i,j}$ in *D* is the transcriptional dissimilarity (*Cost* function) between interpolated point *O_i_* and interpolated point *T_j_*. The *Cost* function in TrAGEDy can be calculated using Euclidean distance, Pearson’s, or Spearman’s correlation. By default, Spearman’s correlation is utilized, with modification so a score of 0 is considered perfect positive correlation:


$$D_{i,j}=\mathrm{Cost}(O_{i},T_{j})$$

where


$$\mathrm{Cost}=\frac{6\sum f_{v}^{2}}{g(g^{2}-1)}$$

and $f_{v}^{2}$ is the difference in ranks of the gene expression values of the interpolated points *O_i_* and *T_j_*, and *g* is the number of genes.

### 2.3 Identifying the optimal path of the two trajectories

To identify the optimal alignment path of the two trajectories, TrAGEDy first identifies the indexes of the minimum start and end points of the alignment, in terms of the dissimilarity score matrix *D* (see Supplementary Fig. S2A for more details):


$$\begin{array}{c} \text{start}=\underset{i}{\text{argmin}}\{\begin{array}{c} D_{i,1}:D_{i,1}<D_{j,1}\;\;\forall j,j\neq i\\ D_{1,i}:D_{1,i}<D_{1,j}\;\;\forall j,j\neq i \end{array}\\ \text{end}=\underset{i}{\text{argmin}}\{\begin{array}{c} D_{i,m}:D_{i,m}<D_{j,m}\;\;\forall j,j\neq i\\ D_{n,i}:D_{n,i}<D_{n,j}\;\;\forall j,j\neq i \end{array} \end{array}$$

Using the minimum dissimilarity value to identify the optimal start and end of the alignment may be misguided in some scenarios. For example, taking the scenario where the optimal starting match of the alignment was $D_{1,2}=0.0001$. This means that any interpolated points that occur before this match are not considered part of the aligned process. The dissimilarity matrix score of $D_{1,1}=0.000101$, thus higher than $D_{1,2}$, but only slightly higher. The dissimilarity between these interpolated points would still be considered very low and thus there is an argument for the alignment starting from $D_{1,1}$ rather than $D_{1,2}$. We thus optimise the possible start and end points by extending towards $D_{1,1}$ and $D_{n,m}$, respectively (Supplementary Fig. S2A and B).

To put a limit on how far towards these points the search goes, we define a cut-off which removes any possible start and end points whose distance, in dissimilarity score from the current start and end points, is greater than the cut-off (Supplementary Fig. S2B):


$$\text{cutOffStart}=\{\begin{array}{cc} \frac{1}{n-1}\sum_{l=2}^{n-1}|D_{l-1,1}-D_{l,1}| & \text{start}=(O_{i},T_{1})\\ \frac{1}{m-1}\sum_{l=2}^{m-1}|D_{1,l-1}-D_{1,l}| & \text{start}=(O_{1},T_{j}) \end{array}$$

$$\text{cutOffEnd}=\{\begin{array}{cc} \frac{1}{n-1}\sum_{l=2}^{n-1}|D_{l-1,m}-D_{l,1}| & \text{end}=(O_{i},T_{m})\\ \frac{1}{m-1}\sum_{l=2}^{m-1}|D_{n,l-1}-D_{n,l}| & \text{end}=(O_{n},T_{j}) \end{array}$$

A variety of possible start and end points for the alignment have thus been identified and their particular costs per match calculated (*paths*). To determine the best scoring path in *paths*, TrAGEDy runs DTW for all the possible start and end point combinations. The final choice of start and end point of the alignment is determined by bootstrapping the cost scores of the paths (Algorithm 1, Supplementary Fig. S2C).

Algorithm 1 Bootstrap cost score of paths

iterateN←UserDefined  ▹ How many times to run bootstrapping

bootstrapLen←min(length(paths))

pathScores←emptyvector

**for** *path* in *paths* **do**

 currentScore←empty vector

 **while**  iterateN≠0  **do**

  append mean(bootstrap(*path*, *bootstrapLen*)) to *currentScore*

  iterateN←iterateN−1

 **end while**

 append *mean*(*currentScore*) to *pathScores*

**end for**

The optimal path through the data (*optimalPath*) is the path in *pathScores* whose average path score is less than the threshold min(pathScores)+mean(S), where *S* is the euclidean distance matrix of mean bootstrapped scores: $S_{f,r}=||\mathrm{pathScore}s_{f}-\mathrm{pathScore}s_{r}|{|}^{2}$

The path whose start and end points are closest to D[1,1] and D[n,m] respectfully, is then chosen as the optimal path.

Having decided on an alignment of the trajectories, TrAGEDy then cuts matches in the alignment which it considers suboptimal, allowing TrAGEDy to deal with the scenario where the biological processes differ in the middle of their respective processes (Supplementary Fig. S3A). Two thresholds are defined. Let *optimalPath* be the indexes of the optimal alignment and let *M* be the indexes of *D* in *optimalPath* and *N* the indexes of *D* not in *optimalPath*:


$$\begin{array}{c} m\mathrm{atchThreshold}=\text{mean}\left(D_{M}\right)\\ n\mathrm{onmatchThreshold}=\text{mean}\left(D_{N}\right) \end{array}$$

We then exclude the indexes of *optimalPath* whose dissimilarity is closer to *nonmatchThreshold* than *matchThreshold*.

### 2.4 Align pseudotime of interpolated points and cells

The aim of aligning the pseudotime of the interpolated points is to give matched interpolated points similar pseudotime values. Interpolated points may only match one another or be a part of a multi-match, where one interpolated point on one of the trajectories is connected to two or more interpolated points on the other trajectory. For adjusting pseudotime of the former scenario [Supplementary Fig. S4A, Equation (1)], given that *O_i_* and *T_j_* are matched and that *T_j_* < *O_i_*:


$$O_{i:n}=O_{i:n}+(T_{j}-O_{i})$$

For a multi-match, first TrAGEDy aligns the pseudotime of the first section of the multimatch [Supplementary Fig. S4B, Equation (2)], using the steps outlined for the individual match, then it does the same for the next match which is not in the multi-match [Supplementary Fig. S4C, Equation (3)]. The interpolated points that are multi-matched to one interpolated point on the other process, have their pseudotime values scaled between the aligned pseudotime value of the first match in the multi-match and the aligned pseudotime value of the match that occurs after the multi-match, adjusted by the difference between pseudotime of the next match and the pseudotime of the first match in the multi-match, normalized by the number of interpolated points being scaled [Supplementary Fig. S4D, Equation (4)]. This process allows the alignment of the interpolated point pseudotime (Supplementary Fig. S4E). TrAGEDy uses the cellAlign method of mapping gene expression values from individual cells onto interpolated points to map pseudotime values from interpolated points onto the individual cells.

### 2.5 Differential expression analysis with TrAGEDy

TrAGEDy identifies DE genes across pseudotime, between the two conditions by taking a sliding window soft clustering approach (Supplementary Fig. S1C). The user defines the number of windows (*nWindow*) of comparison and how much overlap (*overlap*), in terms of matched/multi-matched interpolated points, there is between them. These, and the total number of matches in the alignment (matches=length(optimalPath)), are used to calculate how the interpolated points (and thus the cells) in each match are assigned to the windows of comparison. First, the total number of matches across all of the windows (including overlapping matches) is calculated.


totalMatches=(matches+(overlap×nWindow))−overlap

The number of matches required in each window of comparison is defined using Algorithm 2.

Algorithm 2 Identify number of matches per window

nWindow←User Defined

overlap←User Defined

matches←length(optimalPath)

nWindowDecrease←nWindow

windowList←empty list

**while**  nWindowDecrease≠0  **do**

 currentWindow←round(totalMatches÷nWindowDecrease)

 totalMatches←totalMatches−currentWindow

 append *currentWindow* to *windowList*

 nWindowDecrease←nWindowDecrease−1

Cells are then assigned to interpolated points through a one-round k-means clustering of the pseudotime axis, with cells assigned to the interpolated point which minimizes ${\left(q_{h}-p_{l}\right)}^{2}$, where *q_h_* is the pseudotime value of interpolated point *h* and *p_l_* is the pseudotime of cell *l*.

Statistical comparisons are then carried out for each window based on the cells that are assigned to the interpolated points in that window. A Mann–Whitney *U* test is used to determine significance of the gene expression difference and log_2_FC is calculated using the same method as Seurat V5 (Hao *et al.* 2024).

### 2.6 Data generation and analysis pipelines

The methods used to generate the simulated datasets and carry out the analyses on the simulated, *T. brucei* and T-cell datasets and method comparisons can be found in the paper supplement and the code can be found on the TrAGEDy GitHub page and Zenodo (see ‘Data availability’ section).

## 3 Results

### 3.1 Aligning simulated scRNA-seq datasets

**Figure 1.**
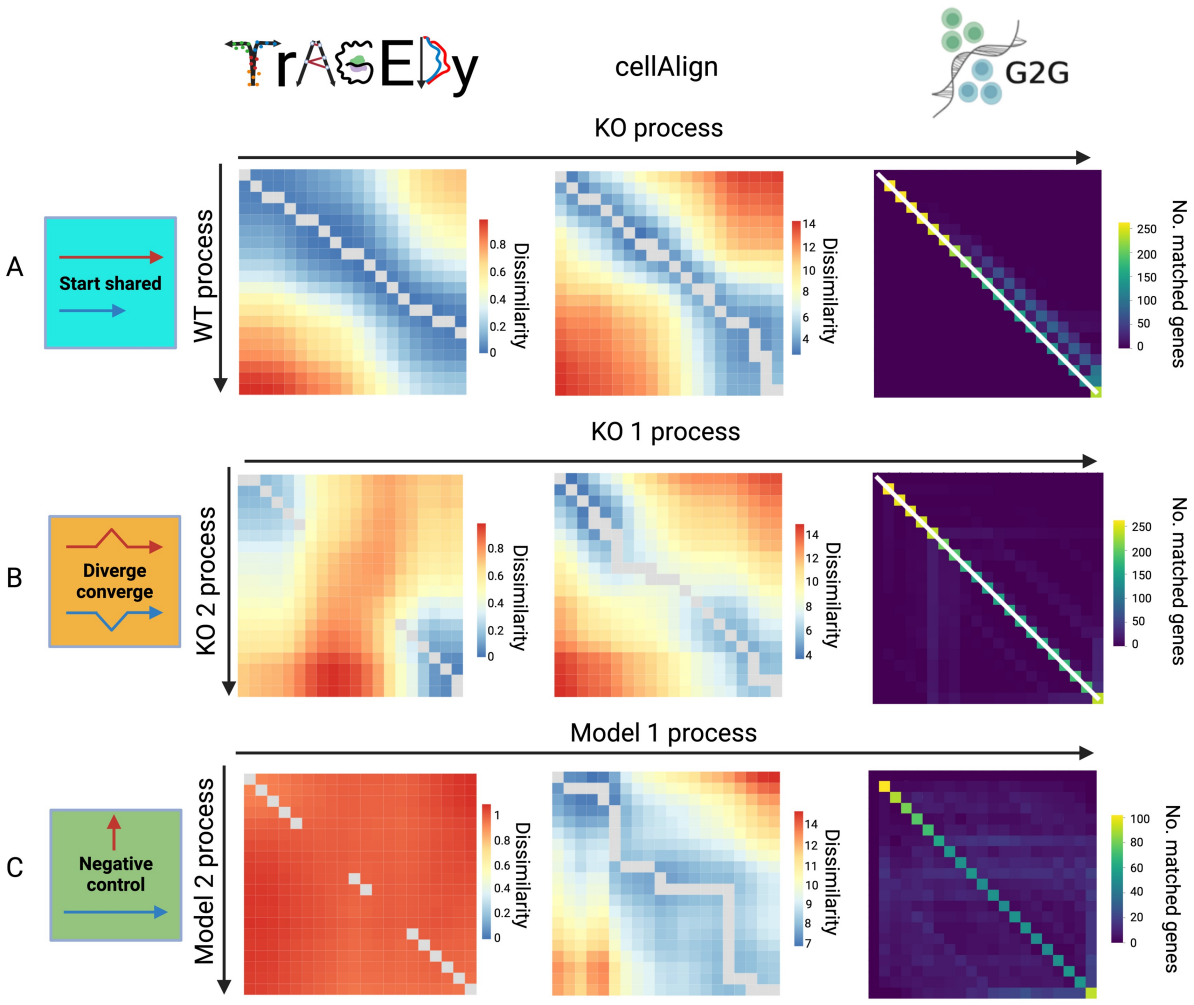
Alignment of simulated single-cell RNA sequencing datasets with TrAGEDy and cellAlign. Heatmaps showing the alignment of the start shared (A), diverge converge (B), and negative control (C). Dyngen simulated datasets as assessed by TrAGEDy, cellAlign, and Genes2Genes (G2G). Each box on the heatmap represents the alignment score of an interpolated point of each of the two datasets. For TrAGEDy and cellAlign, the score represents transcriptional dissimilarity (as assessed by Spearman’s correlation and Euclidean distance, respectively), while G2G shows the number of genes whose expression patterns is matched between the two interpolated points. The grey line represents the path of optimal alignment for TrAGEDy and cellAlign, while in G2G, the optimal alignment is represented by a white line. The alignment heatmap of G2G for the negative control dataset has been edited to remove the alignment line; reflecting its conclusion that the two datasets share no common alignment.

We tested TrAGEDy’s ability to correctly identify the alignment of trajectories on datasets where the alignments were known. Using Dyngen (Cannoodt *et al.* 2021) three different simulated scRNA-seq trajectory dataset pairs were generated: two positive controls and one negative control (Fig. 1A).

The first positive test used two datasets, a full developmental progression involving sequential expression of many genes (wild type; WT), and the second resulting from knockout (KO) of one of these genes (Supplementary Fig. S5). Thus, the KO trajectory is truncated at the point at which the process requires the transcription of the knocked out gene. TrAGEDy correctly aligned these datasets: the WT and the KO datasets initially align with one another, but the KO finishes its full process before the WT. For comparison, we also applied cellAlign and Genes2Genes (G2G), another trajectory alignment method which mainly focuses on aligning single genes but can also be used to align multiple genes at once (Sumanaweera *et al.* 2024). The cellAlign distance matrix revealed the initial alignment of the WT and KO, and the eventual truncation of the KO. However, cellAlign failed to position the correct end point of the KO trajectory relative to the WT, due to running DTW on the whole dissimilarity matrix. G2G also returns an incorrect alignment, suggesting that the simulated WT and KO datasets have a 1:1 alignment (Fig. 1A).

To assess how the methods handle a scenario where the trajectories have shared start and end points, but deviate in the middle, we simulated two datasets with Dyngen that have a diverge-converge backbone (Supplementary Fig. S5). TrAGEDy accurately captured the initial and terminal alignment of the two trajectories, leaving the middle sections unaligned. CellAlign failed to align the datasets correctly, as it does not include functionality to prune matches. G2G also returned an incorrect alignment, as it suggested that there was a 1:1 alignment between the two simulated KO datasets (Fig. 1B).

For the negative control, two datasets with distinct gene regulatory networks and transcription kinetics were generated (referred to as Model 1 and Model 2) (Supplementary Fig. S5). As there is no shared process between the conditions, the dissimilarity is expected to be high and no alignment should be found. The dissimilarity score calculated by TrAGEDy showed that most of the matches have a dissimilarity score close to 1, and thus do not share a common process. As TrAGEDy finds the optimal path by looking at the context of the whole dissimilarity matrix, even if the dissimilarity scores are high, it will still find a path (Fig. 1C). TrAGEDy will give a warning to the user if the median dissimilarity score of the path is high, thus it is up to the user to interpret the resulting alignment in the context of the overall score of the path. CellAlign also creates a path through the dissimilarity matrix, which has a higher overall dissimilarity range than the other two datasets, with the Euclidean distance ranging from ∼7 to 14 (Fig. 1C), while the other two datasets ranged from ∼4 to 14 (Fig. 1A and B). If Pearson correlation is used for cellAlign, the alignment heatmap shows little transcriptional similarity between the datasets (Supplementary Fig. S6C). As G2G directly models insertion and deletions, it can be determined that there is no common alignment between the two datasets (Fig. 1D).

As of 2019, over 70 TI methods have been published, all with varying degrees of performance (Saelens *et al.* 2019). Given the diversity of TI methods, and their performance, we tested whether TrAGEDy returns similar results for the simulated datasets using different pseudotime methods. Pseudotime values were generated for the simulated datasets using Monocle 3 (Cao *et al.* 2019) and PAGA (Wolf *et al.* 2019), and used as the basis for performing TrAGEDy. The results show that across different TI methods, TrAGEDy still returns the accurate underlying alignment for the simulated scRNA-seq datasets (Supplementary Fig. S7).

These analyses show that TrAGEDy is capable of faithfully capturing the underlying alignments of simulated biological scRNA-seq datasets, across a variety of TI methods.

### 3.2 WT vs ZC3H20 KO *Trypanosoma brucei* alignment

Simulated data represents a ‘best case’ scenario for benchmarking tools, and often lacks the complexity of real datasets. We thus decided to apply TrAGEDy to real scRNA-seq datasets where the underlying processes have been characterized. For our first application, we examined a distinct developmental process in the kinetoplastid parasite *T. brucei*. Bloodstream form *T. brucei* which are null for the RNA binding protein *ZC3H20*, are unable to undergo the transition between their Long Slender (LS) and Short Stumpy (SS) stages (Cayla *et al.* 2020, Liu *et al.* 2020) and fail to express stumpy associated genes. Briggs *et al.* (2021) used 10x Chromium to sequence two biological replicates of WT *T. brucei* parasites (WT01 and WT02) undergoing this transition *in vitro*, as well as *ZC3H20* KO failing to differentiate in the same conditions. The authors defined four main clusters, LS A and B, and SS A and B, to show the progression of the transition, with LS A being the start and SS B being the end. Thus, the final alignment is expected to show that the WT and the *ZC3H20* KO datasets share an initial common developmental process, but that the KO process is truncated or branched relative to the WT, as suggested by previous analysis using data integration and Slingshot TI (Street *et al.* 2018).

**Figure 2.**
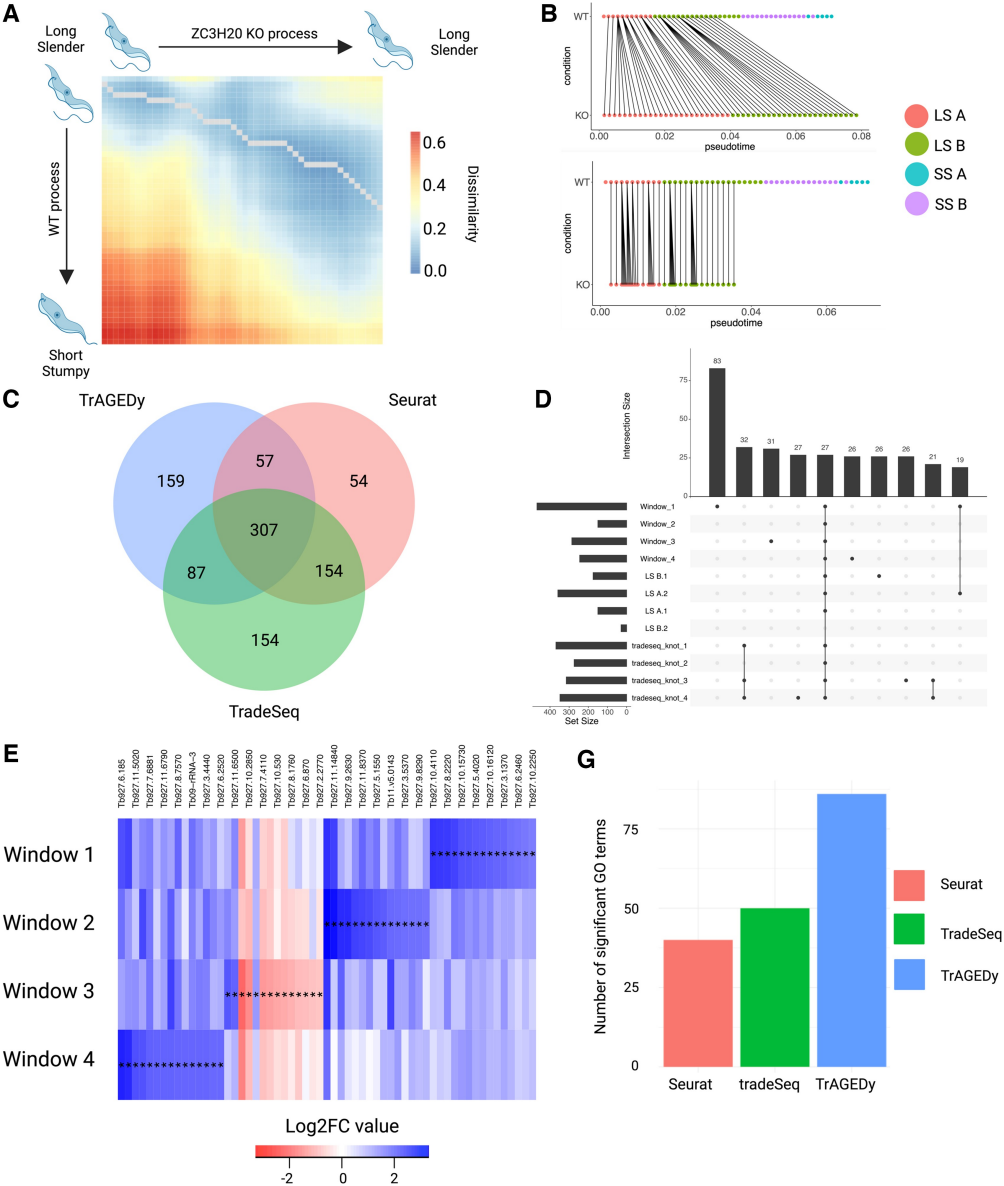
Analysis of WT and *ZC3H20* KO *Trypanosoma brucei* development with TrAGEDy. TrAGEDy alignment of the WT and *ZC3H20* KO trajectories of *T. brucei* development with transcriptional dissimilarity calculated with Spearman correlation (A). WT and *Bcl11b* KO trajectories before and after alignment with TrAGEDy, showing connections between the two processes (B). Venn diagram showing the intersection of the DE genes captured by TrAGEDy, TradeSeq, and Seurat (C). UpSet plot showing the top 10 intersections of DE genes captured by TrAGEDy across four windows of comparison and Seurat over four clusters and TradeSeq over four knot comparisons (D). Heatmap showing DE genes with the highest Log_2_FC captured (but not uniquely captured) between the WT and *ZC3H20* KO trajectories at four different windows of comparison. Each index is coloured by the Log_2_FC and a * symbol indicates the difference is significant [Bonferroni adjusted *P* < .05, Mann–Whitney *U* test, abs(Log_2_FC) > 0.5, minimum percentage expressed > 0.1]. A full list of heatmap genes can be found in Supplementary File S6 (E). Barplot showing the number of significant Gene Ontology (GO) terms (Benjamini–Hochberg adjusted *P*-value < .01) returned when GO enrichment analysis was carried out using TriTrypDB on all the DE genes returned by TrAGEDy, TradeSeq, and Seurat (F).

PHATE (Moon *et al.* 2019) embeddings were generated independently for the three datasets: two WT replicates (WT01 and WT02) and the *ZC3H20* KO. The PHATE embeddings were used as the basis for Slingshot TI to calculate pseudotime values for each of the cells. Applying TrAGEDy to WT01 and WT02 returned an alignment that started at the same point, and ended just before the final endpoint, returning a nearly 1:1 alignment, as predicted of biological replicates (Supplementary Fig. S8). TrAGEDy alignment of the WT replicates and the *ZC3H20* KO dataset show that the two processes have an initial point of alignment but the *ZC3H20* KO process was truncated relative to the WT (Fig. 2A). The trajectory pseudotime values were then adjusted using the TrAGEDy calculated path (Fig. 2B), with the pseudotime of the KO cells being reduced post-TrAGEDy alignment as expected (Supplementary Fig. S9).

By aligning trajectories rather than integrating the datasets, we expected to preserve more variability, which may be reflected in the DE genes captured. Therefore, we compared TrAGEDy with other methods that can extract DE genes between conditions. We compared TrAGEDy’s DE detection ability against established methods, namely Seurat V5 (Hao *et al.* 2024) and TradeSeq (Van den Berge *et al.* 2020).

TrAGEDy detected more significant DE genes than both Seurat and TradeSeq (Fig. 2C and D), uniquely capturing 159 genes as DE, while TradeSeq and Seurat only returned 154 and 54, respectively (Fig. 2C, Supplementary Files S1–S3). Plotting the smoothed expression of some of the DE genes unique to TrAGDEy showed that the expression patterns match what TrAGEDy returns (Fig. 2E, Supplementary Fig. S11). In terms of runtime required to return DE results, both TrAGEDy and Seurat returned results in under a minute, while TradeSeq took around 2 h (Supplementary File S7). Returning more significant DE genes is not a robust metric for assessing how well a DE gene detection method is performing. As an example, randomly assigning 75% of the genes in the dataset to be DE result in more DE genes returned than most DE gene detection methods. Furthermore, there is no direct ground truth for whether the genes captured are DE or not. To address these issues, we used the number of significant GO terms returned from each DE gene list as a proxy for how biologically meaningful the output of the DE gene detection methods is. This approach was previously utilized in the article describing PseudotimeDE (Song and Li 2021). Biological process GO term analysis was run on the three methods’ returned DE genes. TrAGEDy returned 86 significant GO terms compared to 50 from TradeSeq and 40 from Seurat (Fig. 2G) (Supplementary File S4), with all but two of the GO terms returned by Seurat also returned by TrAGEDy and only six GO terms being unique to TradeSeq (Supplementary Fig. S10B). When GO terms were generated on the list of uniquely captured genes for each method, only TrAGEDy returned any significant GO terms (Supplementary Fig. S10A, Supplementary File S5).

The above results show that TrAGEDy reveals more biologically relevant information on the LS to SS transitions under WT and *ZC3H20* KO conditions than established protocols.

### 3.3 WT vs Bcl11b KO T-cell development analysis

With the next application, we used TrAGEDy in an analysis scenario which has not been attempted. For this, we applied TrAGEDy to a scRNA-seq dataset of *in vitro* T-cell development in WT and *Bcl11b* KO mice over two timepoints (Zhou *et al.* 2022). The data contain cells undergoing the early stages of T-cell development in the thymus, from thymus-seeding precursors (TSP) to around the Double negative (DN) 4/Double positive (DP) stage. The authors find that when *Bcl11b* expression is knocked out, the cells deviate from the T-cell commitment pathway and develop distinct transcriptomic signatures around the DN2 stage of T-cell development. However, some *Bcl11b* KO cells still express markers of T-cell pathway commitment, such as the gene for the pre-T-cell Antigen receptor *α* (*Ptcra*) (Hwang *et al.* 2020) and *Rag1* (Supplementary Fig. S12), one of the central genes responsible for T-cell receptor gene recombination, suggesting there may be some KO cells that have reached the post DN2 stages where the cells express a pre-TCR receptor and undergo *β*-selection (Mombaerts *et al.* 1992). In this context, we aimed to use TrAGEDy to compare the WT and *Bcl11b* KO trajectories and identify differences in transcriptomes that occur between them as they continue down the normal path of T-cell development.

**Figure 3.**
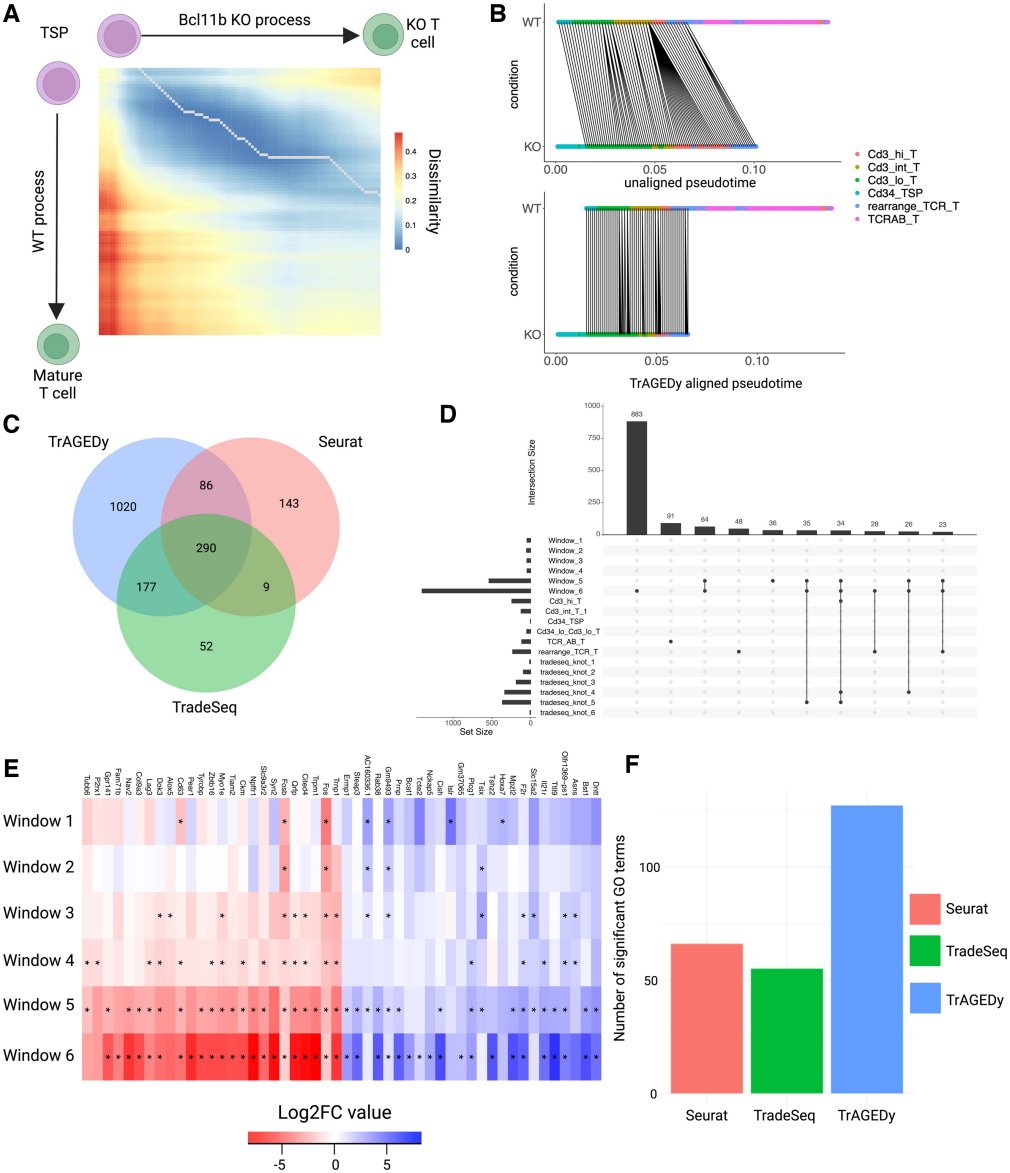
Analysis of WT and *Bcl11b* KO trajectories of T-cell development with TrAGEDy. TrAGEDy alignment of the WT and *Bcl11b* KO trajectories of T-cell development with transcriptional dissimilarity calculated with Spearman correlation (A). WT and *Bcl11b* KO trajectories before and after alignment with TrAGEDy, showing connections between the two processes (B). Venn diagram showing the intersection of the DE genes captured by TrAGEDy, TradeSeq, and Seurat (C). UpSet plot showing the top 10 intersections of DE genes captured by TrAGEDy across six windows of comparison and Seurat over six clusters and TradeSeq over six knot comparisons (D). Heatmap showing DE genes captured *Bcl11b* KO trajectories at six different windows of comparison (as in Fig. 3). Full list of genes can be found in Supplementary File S13 (E). Barplot of GO terms of all the DE genes returned by TrAGEDy, TradeSeq, and Seurat (F).

The cells were sequenced over two runs, with each run containing a variety of biological and technical replicates, and sampling days. For simplicity, WT1 and WT2 denote the two sequencing runs of WT cells, while KO1 and KO2 denote the two sequencing runs of *Bcl11b* KO cells. When projected into the PHATE space, some of the WT1 cells were separated from the main body of the trajectory, which had downstream effects on the alignment, and so these cells were removed because TrAGEDy cannot function when there are gaps in the pseudotime axis (Supplementary Fig. S13). TrAGEDy first aligned the two datasets for each condition, resulting in two separate alignments: a WT alignment and a *Bcl11b* KO alignment (Supplementary Fig. S14). The WT1 dataset only contains cells from day 10 of sampling, while WT2 contains cells from days 10 and 13. TrAGEDy captures this difference, showing a strong initial alignment of the two WT datasets with the WT1 dataset finishing before the WT2 (Supplementary Fig. S14A). TrAGEDy was then performed on the aligned WT and *Bcl11b* KO trajectories. The resulting final alignment shows the two conditions sharing an initial common process, but the *Bcl11b* KO developmental progression finishes around the point when cells transition to express both TCR *α* and *β* chain genes (Fig. 3A and B). Viewing the PHATE space of the individual sequencing runs, coloured by the pre- and post-TrAGEDy pseudotimes, shows the truncation of the *Bcl11b* KO cells across the pseudotime axis (Supplementary Fig. S15).

Overall, TrAGEDy returned 1573 DE genes across 6 windows of comparison, while TradeSeq and Seurat captured 528 DE genes across 6 knots and 6 cluster comparisons, respectively (Fig. 3C, Supplementary Files S8–S10). All the methods found more DE genes towards the end of the biological process compared with the beginning (Fig. 3D). For genes only captured by one of the methods, TrAGEDy uniquely captured 1020 DE genes, while Seurat returned 143 and TradeSeq 52 (Fig. 3C, Supplementary Files S8–S10). Plotting some of the DE genes that only TrAGEDy captured with TradeSeq smoothed expression show they are concurrent with the patterns of expression TrAGEDy suggests (Fig. 3E, Supplementary Fig. S17). The times taken to return DE results was longer than the *T. brucei* analysis for all methods, however, Seurat still managed to return results within seconds and TrAGEDy only took just over 2 min. In contrast, TradeSeq took over 20 h to return a result (Supplementary File S7).

TrAGEDy found the most significant GO terms compared to Seurat and TradeSeq (Fig. 3G, Supplementary File S11). Furthermore, when only the uniquely captured DE genes were utilized, TrAGEDy was the only method that returned any significant GO terms (Supplementary Fig. S16A, Supplementary File S12). Of the significant GO terms returned by the methods for their entire DE gene list, only 15 were shared between all three methods (Supplementary Fig. S16B).

## 4 Discussion

In this article, we present TrAGEDy, a tool for aligning and comparing single-cell trajectories between conditions. Across multiple simulated datasets, TrAGEDy can find the underlying alignment between two processes, where cellAlign and G2G fails, due to its ability to prune matches and identify optimal start and end points in the dissimilarity matrix (Fig. 1A). Across biological replicates and different conditions of real datasets, TrAGEDy overcomes batch effects to deliver accurate alignments and identify biologically relevant genes.

In almost all of the simulated datasets discussed here, TrAGEDy was able to correctly align simulated trajectories with greater accuracy than cellAlign and G2G. For cellAlign, the constraints of DTW hamper it from accurately capturing the underlying alignments, while for G2G it may be due to the fact that the final alignment is based on an aggregate of single gene alignments, rather than directly looking at the global alignment of genes across the cells. Additionally, TrAGEDy alignment of real data sets resulted in expected outcomes, including the complete alignment of biological replicates and partial alignments between conditions.

To compare DE results of TrAGEDy we also performed differential gene expression tests using two widely used tools: Seurat and TradeSeq. Although Seurat does not take pseudotime into account when it performs differential expression tests it is possible to compare cells across conditions, something which is not possible in many tools designed to specifically analyse trajectories, including pseudotimeDE and Monocle (Trapnell *et al.* 2014, Song and Li 2021). Low detection of DE genes by Seurat relative to TrAGEDy could be explained by the fact Seurat does not take pseudotime into account when carrying out the differential expression tests. The biological interpretation of the DE genes detected only by TrAGEDy also fits with the biological processes of the *T. brucei* and T-cell alignments, this is discussed in the paper supplement.

In terms of runtime, Seurat performed the best due to optimization through the Presto algorithm (Korsunsky *et al.* 2019), but TrAGEDy was not far behind in terms of runtime and performed better than Seurat in terms of the results returned. The main bottleneck for TradeSeq runtime was fitting the GAMs to each gene. TrAGEDy thus serves as a better alternative requiring less time to run, less resources and provides more biological insightful results than TradeSeq.

Ultimately, TI represents an approximation of how a developmental trajectory might look and may not represent the actual ordering of the cells through the process. This is confounded by distortions in cell relationships induced by dimensionality reduction methods (Chari and Pachter 2023). Adding extra temporal information from lineage tracing will help add more biological validity to single-cell data (Wagner and Klein 2020). TrAGEDy could thus take lineage trace-inferred time, rather than pseudotime, as its input to allow the analysis of such datasets, allowing it to remain relevant as more sophisticated single-cell sequencing techniques are introduced. Furthermore, the advent of perturb-seq allows researchers to more easily generate and analyse the effect of genetic perturbations on processes (Schraivogel *et al.* 2020), providing multiple and diverse opportunities for TrAGEDy to be applied.

## 5 Conclusion

Our paper demonstrates the ability of TrAGEDy to overcome batch effects between datasets, without the need for data integration, and to reveal the underlying alignment between datasets; identifying shared and dissimilar points across the independent trajectories. With its alignments, TrAGEDy overcomes constraints of current tools, which limit the alignment to datasets which may start and end at different points or differ in the middle. TrAGEDy can identify more DE genes than other methods, and in all cases, identify more biologically relevant processes enriched in the different conditions. With the advent of perturb-seq methods, the need for TrAGEDy will become more apparent and with lineage tracing it will make the time axis more biological accurate. Thus, this tool increases the capacity to analyse developmental progression using scRNA-seq to reveal meaningful insights into the shared biological processes.

## Supplementary Material

btaf073_Supplementary_Data

## Data Availability

The processed Dyngen simulated RDS and *T. brucei* RDS objects are available through the Zenodo (doi: 10.5281/zenodo.13310931). The processed RDS object of the *Bcl11b* KO and WT T cells was obtained from the corresponding author of the Zhou *et al.* (2022) paper. The code used to generate these results can be found on the TrAGEDy GitHub page (https://github.com/No2Ross/TrAGEDy) and through Zenodo (doi: 10.5281/zenodo.13310931).
