# Supplementary material for: TrAGEDy—trajectory alignment of gene expression dynamics
Source: Bioinformatics. 2025 Mar 11;41(3):btaf073. doi: 10.1093/bioinformatics/btaf073 (PMC11908647; doi:10.1093/bioinformatics/btaf073)
Supplement: btaf073_Supplementary_Data [file btaf073_supplementary_data.pdf]

1 Supplementary methods

1.1 Simulating trajectories with Dyngen

All simulated datasets were simulated with Dyngen (Cannoodt et al. 2021). Each simulated dataset contains 1500 cells with 150 housekeeping genes, 250 target genes and a number of transcription factors equal to the number specified by the backbone of the model.

The first positive control experiment trajectories were simulated with a linear backbone with parameters drawn from the same transcription factor, feature and kinetic network distributions. One of the datasets was not modified beyond the standard pipeline, while the other had a full knockout in the B5 gene module, causing a stunted process in comparison to the other dataset.

The second positive control experiment trajectories were simulated with a bifurcating-converging backbone with parameters drawn from the same transcription factor, feature and kinetic network distributions. One of the datasets had a full knockout in the C1 gene module while the other dataset had a full knockout in the D1 gene module.

The negative control experiment trajectories were simulated with a linear backbone with parameters drawn from different transcription factor, feature and kinetic network distributions.

1.2 Applying TrAGEDy, Genes2Genes and cellAlign to simulated trajectories

All simulated datasets were processed using the following. In Seurat, the simulated counts were normalized prior to scaling across all simulated genes. Principal Component Analysis (PCA) was performed on the scaled data and using the first 5 PCA dimensions clustering was performed at a resolution of 0.2 for all the datasets as well as UMAP. Slingshot (Street et al. 2018) was performed to get pseudotime values for each of the cells, using the UMAP space as the basis for the trajectory. To get the feature space, the top 100 DE genes in terms of Log_2FC for each cluster in each dataset were collected for both datasets being compared. The prior analysis steps were kept the same for both cellAlign and TrAGEDy analysis, as well as the window size and the number of interpolated points, with a window size equal to the maximum pseudotime divided by 18 and the number of interpolated points being 20. For cellAlign, Euclidean distance and Pearson correlation was used to calculate dissimilarity, while for TrAGEDy Spearman correlation was used. For Genes2Genes, the pseudotime was scaled between 0 and 1 and a binning number of 20 was used for all the comparisons and the same feature space was used as in the TrAGEDy and cellAlign analyses.

TrAGEDy was also applied to the same simulated datasets, with pseudotime generated using Monocle 3 and PAGA. PAGA pseudotime was generated by first analysing the simulated dataset using Scanpy. Briefly, this involved performing PCA on the scaled, normalised expression values of the datasets and using the first five PCs to calculate the 20 nearest neighbors for the cells. PCA was only done on the genes that were included in the previously identified feature space. The nearest neighbor graph was then used as the basis for Leiden clustering (resolution = 0.2). PAGA was then run, using the Leiden cluster centroids as the nodes, and the data further reduced into two diffusion components, which were used to calculate the pseudotime of the cells.

Monocle 3 pseudotime was calculated by first creating a CellDataSet (CDS) object and performing PCA on the log and size factor normalised expression data. Only genes that were present in the previously identified feature space were utilised for PCA. The top four PCs were used as the basis for UMAP and Leiden clustering (resolution = 1×10^{-5}), with the UMAP space being used as the basis for Monocle 3 to assign pseudotime values to the cells.

1.3 Pre-processing of WT vs *ZC3H20* KO *Trypanosoma brucei* dataset

The processed RDS (R dataset) file containing the final cluster labels was acquired from the authors. The 'LS A.1' and 'LS A.2' clusters were merged into the cluster 'LS A' and the 'LS B.1' and 'LS B.2' were merged into the cluster 'LS B'.

To ensure comparability of the metrics returned, the $\log_2$FC of the genes between conditions was calculated for TrAGEDy, Seurat V5 and TradeSeq using the formula used in Seurat V5 (Y. Hao, Stuart, et al. 2024).

1.4 TrAGEDy analysis of WT vs *ZC3H20* KO *Trypanosoma brucei* dataset

The integrated object was split into the individual datasets (WT01, WT02 and *ZC3H20*_KO). The top 200 DE genes (defined as absolute Log_2FC > 0.75, Bonferroni corrected p-value < 0.05 and the gene expressed in at least 25% of cells) across the clusters were found for each dataset and used as the basis for constructing the PHATE space and assessing similarity with TrAGEDy. The number of genes in the feature space amounted to 518 genes. PHATE embeddings were constructed from the normalised gene expression count matrix. Pseudotime was calculated using Slingshot for each of the three datasets, with the 'LS A' cluster used as the starting point. The WT01 and WT02 datasets were then aligned with TrAGEDy, with 50 interpolated points used to align the processes, with

a window size of the maximum pseudotime value, from either of the two datasets being analysed, divided by 45. This TrAGEDy aligned WT dataset was then aligned against the *ZC3H20* KO dataset. Differential expression was carried out over 4 windows, with a gene identified as being DE if the absolute Log_2FC was more than 0.5, Bonferroni corrected p-value < 0.05 and the gene expressed in at least 10% of cells in one of the conditions. The final TrAGEDy aligned WT and *ZC3H20* KO datasets were then analysed independently by TradeSeq to create the smooth gene expression plots across the TrAGEDy aligned pseudotime.

1.5 Seurat analysis of WT vs *ZC3H20* KO *Trypanosoma brucei* dataset

The 'SS A' and 'SS B' clusters were removed from the integrated dataset, as they are not present in the *ZC3H20* KO dataset, and the FindMarkers function was performed for all the four slender clusters (LS A.1, LS A.2, LS B.1 & LS B.2) between the WT and *ZC3H20* KO conditions. A gene identified as being DE if the absolute Log_2FC was more than 0.5, Bonferroni corrected p-value < 0.05 and the gene expressed in at least 10% of cells in one of the conditions.

1.6 TradeSeq analysis of WT vs *ZC3H20* KO *Trypanosoma brucei* dataset

The 'SS A' and 'SS B' clusters were removed from the integrated dataset. Using the authors PHATE space, Slingshot was applied to build trajectories. The optimal number of knots for fitting the general additive model to the dataset was assessed through the evaluateK function, with the optimal number determined to be 8. FitGAM was run on the dataset followed by the conditionTest function to identify DE genes between the WT and *Bcl11b* KO conditions. 4 knot comparisons were made, and a gene were determined to be DE if the absolute Log_2FC was greater than 0.5, Bonferroni p-value < 0.05 and the gene was expressed in at least 10% of the cells that fell within the current knot comparison, in one of the conditions.

For all cases, genes were analysed in TriTrypDB to get their gene short names and functions (Amos et al. 2022, Alvarez-Jarreta et al. 2024).

1.7 Preprocessing of WT vs *Bcl11b* KO *in vitro* T cell development dataset prior to TrAGEDy analysis

The RDS (R dataset) file containing the scRNA-seq information was acquired from the authors (Zhou et al. 2022).

The dataset was split into the WT and *Bcl11b* KO conditions and then further split into the different sequencing runs. For each of the four scRNA-seq objects derived from the previous, the data was analysed using the Seurat V5 pipeline. When the data was scaled, the effects of the cell cycle were regressed out by passing the cell cycle associated genes Seurat provides into the var.to.regress parameter. For each dataset, PCA was carried out on the scaled-regressed expression matrix, reducing the dimensions down to 50. An elbow plot was used to determine the number of Principal Components (PC) used to create a UMAP space embedding (WT1 = 22, WT2 = 20, KO1 = 25, KO2 = 30). Clustering was performed using the same number of PCs for UMAP embedding calculation. Clustree (Zappia and Oshlack 2018) was used to help choose a resolution for clustering that leads to stable clusters. Clusters were annotated as follows. Clusters that did not express Cd3 genes but expressed Cd34 were defined as 'Cd34_TSP', clusters which did not express Cd34 or Cd3 were characterized as 'early_T', clusters which had low expression of Cd3 but no Cd34 expression were characterised as 'Cd34_lo_Cd3_lo_T', those with high Cd3 expression were 'Cd3_T_hi' and those with middle levels of Cd3 expression were 'Cd3_T_mid'. Clusters which had high expression of Cd3 and expressed *Ptcra* and *Rag1* were defined as 'rearrange_TCR_T' and those that expressed the TCR signal transduction molecule *Zap70* and some cells that express the gene *Trac*, were defined as 'TCR_AB_T'. Clusters that express *Rora* were defined as 'Rora_T', clusters that expressed high levels of interferon associated genes were defined as 'Interferon_response' and cells which clustered out with the main body of the UMAP were classed as 'Outlier'. The 'Interferon_response' and 'Outlier' clusters were removed before downstream analysis with TI, TrAGEDy, Seurat DE or TradeSeq.

1.8 TrAGEDy analysis of WT vs *Bcl11b* KO *in vitro* T cell development dataset

In order to build a gene feature space to construct PHATE embeddings and assess dissimilarity with TrAGEDy, the feature space for the dataset was made of the combined DE genes for each cluster whose absolute $Log_2FC >$ 0.5, Bonferroni corrected p-value < 0.05 and was expressed in at least 1% of cells across all four of the datasets. To reduce the effect of the cell cycle, genes supplied by Seurat as being important in the cell cycle and genes included in the Mouse Genome Informatics GO term 'cell cycle' were removed from the feature space. For all four of the individual datasets, 10 dimensional PHATE embeddings were generated from the normalised gene expression count matrix for the features selected previously. For the WT1 dataset, cells with a pseudotime higher than 0.1 were removed. TI was then carried out using Slingshot on the PHATE embeddings with the Cd34_TSP cluster chosen as the starting point. TrAGEDy was carried out on each of the conditions, using 100 interpolated points with a window size of the maximum pseudotime value, from either of the two datasets being analysed, divided by 90. TrAGEDy aligned pseudotime values were then created, resulting in a TrAGEDy aligned WT and a TrAGEDy aligned *Bcl11b* KO dataset.

The WT and *Bcl11b* KO TrAGEDy aligned datasets were then analysed using TrAGEDy. TrAGEDy was carried out with the same number of interpolated points and window size as the replicate alignments. TrAGEDy differential expression test was then carried out across 6 windows of comparison. A gene was returned as being differentially expressed if the absolute Log_2FC was greater than 0.75, Bonferroni adjusted p-value < 0.05 and the gene was expressed in at least 10% of cells in the window of comparison, in one of the conditions. The final TrAGEDy aligned WT and *Bcl11b* KO datasets were then analysed independently by TradeSeq to create the smooth gene expression plots across the TrAGEDy aligned pseudotime.

1.9 Seurat analysis of WT vs *Bcl11b* KO *in vitro* T cell development dataset

Using Seurat V5 integration (Satija et al. 2015, Butler et al. 2018, Stuart et al. 2019, Y. Hao, S. Hao, et al. 2021 and Y. Hao, Stuart, et al. 2024), the four datasets were integrated using the 'integrated.cca' method. When the data was scaled, the effects of the cell cycle were regressed out by passing the cell cycle associated genes Seurat provides into the var.to.regress parameter. PCA was carried out on the scaled-regressed expression matrix, reducing the dimensions down to 50. An elbow plot was used to determine the number of Principal Components (PC) used to create a UMAP space embedding. Clustering was performed using the same number of PCs for UMAP embedding calculation. Clustree was used to help choose a resolution for clustering that leads to stable clusters and clusters were annotated as described previously. The FindMarkers function was used to identify DE genes between the conditions for each cluster. Genes were determined to be DE if the absolute Log_2FC was greater than 0.75, Bonferroni adjusted p-value < 0.05 and the gene was expressed in at least 10% of the cells in one of the conditions for the cluster being compared.

1.10 TradeSeq analysis of WT vs *Bcl11b* KO *in vitro* T cell development dataset

Using the Seurat integrated, cell cycle regressed dataset; PHATE was carried out, using the scaled and cell cycle regressed gene expression matrix. Slingshot was then applied to the dataset, using the Cd34_TSP cluster as the starting point of the trajectory. Cells on the trajectory path which ended with the TCR_AB_T cluster was kept and all other cells were removed from the dataset. The optimal number of knots for fitting the general additive model to the dataset was assessed through the evaluateK function, with the optimal number determined to be 7. FitGAM was run on the dataset followed by the conditionTest function to identify DE genes between the WT and *Bcl11b* KO conditions. 6 different knot comparisons were made, and a gene were determined to be DE if the absolute Log_2FC was greater than 0.75, Bonferroni p-value < 0.05 and the gene was expressed in at least 10% of the cells that fell within the current knot comparison, in one of the conditions.

1.11 GO term analysis of T cell DE genes

Biological process GO term analysis of the lists of DE genes (either unique DE genes or all of them) for the three methods was carried out using the enrichGO command of clusterProfiler. Background genes were the genes whose expression was more than 5% across the entire integrated T cell dataset. GO terms whose Benjamini-Hochberg corrected p-value was less than 0.01 were identified as significantly enriched GO terms.

1.12 GO term analysis of *Trypanosoma brucei* DE genes

Biological process GO term analysis of the lists of DE genes (either unique DE genes or all of them) for the three methods was carried out using TriTrypDB. GO terms whose Benjamini-Hochberg corrected p-value was less than 0.01 were identified as significantly enriched GO terms.

1.13 Runtime experiments

Packages were run single threaded on a virtual machine with 70 gigabytes of allotted RAM and 3 GHz clock speed. Runtime experiment code detailing what sections were included for each package when calculating runtime can be found on our GitHub.

1.14 Package versions details

All analysis and experiments were carried out on R were done with version 4.1.2 except for the Dyngen dataset simulation and the TradeSeq experiments which were carried out on R version 4.1.0. Seurat version 5.0.3, Dyngen version 1.0.5, phateR version 1.0.7, Slingshot version 2.2.1, Single Cell Experiment version 1.16.0, cellAlign version 0.1.0, matrixStats version 1.1.0 & TradeSeq version 1.6.0 were used.

For the Genes2Genes experiments, analysis was done on Python version 3.8.16 with the following package versions: genes2genes version 0.2.0, numpy version 1.24.3, pandas version 2.0.3, scanpy version 1.9.3, scipy version 1.10.1, matplotlib version 3.7.1, seaborn version 0.12.2 & scikit-learn version 1.2.2.

2 Supplementary files

Supplementary file 1 - TrAGEDy DE results across four windows of comparison for the TrAGEDy aligned WT vs *ZC3H20* KO *T. brucei* trajectories. Significant DE genes are identified as those with an absolute $Log_2FC > 0.5$, bonferroni adjusted p-value less than 0.05 and were expressed in at least 10% of cells in one of the conditions in the window.

Supplementary file 2 - Tradeseq DE results across four knots of comparison for the TrAGEDy aligned WT vs *ZC3H20* KO *T. brucei* trajectories. Significant DE genes are identified as those with an absolute $Log_2FC > 0.5$, bonferroni adjusted p-value less than 0.05 and were expressed in at least 10% of cells in one of the conditions in the window.

Supplementary file 3 - Seurat DE results across four clusters of comparison for the TrAGEDy aligned WT vs *ZC3H20* KO *T. brucei* trajectories. Significant DE genes are identified as those with an absolute $Log_2FC > 0.5$, bonferroni adjusted p-value less than 0.05 and were expressed in at least 10% of cells in one of the conditions in the window.

Supplementary file 4 - Significant (Benjamini p-value less than 0.05) GO terms, generated using TriTrypDB, for all the significant DE genes captured by TrAGEDy, tradeSeq and Seurat for the WT vs *ZC3H20* KO *T. brucei* comparison.

Supplementary file 5 - Significant (Benjamini p-value less than 0.05) GO terms, generated using TriTrypDB, for the significant DE genes uniquely captured by TrAGEDy, tradeSeq and Seurat for the WT vs *ZC3H20* KO *T. brucei* comparison.

Supplementary file 6 - Genes IDs and associated Log_2FC for the heatmap in figure 3E.

Supplementary file 7 - Table of the runtimes (in minutes) taken by TrAGEDy, Seurat and TradeSeq to return DE results for the *T. brucei* and T cell datasets.

Supplementary file 8 - TrAGEDy DE results across four windows of comparison for the TrAGEDy aligned WT vs *Bcl11b* KO T cell trajectories. Significant DE genes are identified as those with an absolute $Log_2FC > 0.75$, bonferroni adjusted p-value less than 0.05 and were expressed in at least 10% of cells in one of the conditions in the window.

Supplementary file 9 - Tradeseq DE results across four knots of comparison for the TrAGEDy aligned WT vs *Bcl11b* KO T cell trajectories. Significant DE genes are identified as those with an absolute $Log_2FC > 0.75$, bonferroni adjusted p-value less than 0.05 and were expressed in at least 10% of cells in one of the conditions in the window.

Supplementary file 10 - Seurat DE results across four clusters of comparison for the TrAGEDy aligned WT vs *Bcl11b* KO T cell trajectories. Significant DE genes are identified as those with an absolute $Log_2FC > 0.75$, bonferroni adjusted p-value less than 0.05 and were expressed in at least 10% of cells in one of the conditions in the window.

Supplementary file 11 - Significant (Benjamini p-value less than 0.05) GO terms, generated using clusterProfiler, for all the significant DE genes captured by TrAGEDy, tradeSeq and Seurat for the WT vs *Bcl11b* KO T cell comparison.

Supplementary file 12 - Significant (Benjamini p-value less than 0.05) GO terms, generated using clusterProfiler, for all the uniquely captured DE genes by TrAGEDy, tradeSeq and Seurat for the WT vs *Bcl11b* KO T cell comparison.

Supplementary file 13 - Genes IDs and associated Log_2FC for the heatmap in figure 4E.

3 Supplementary figures

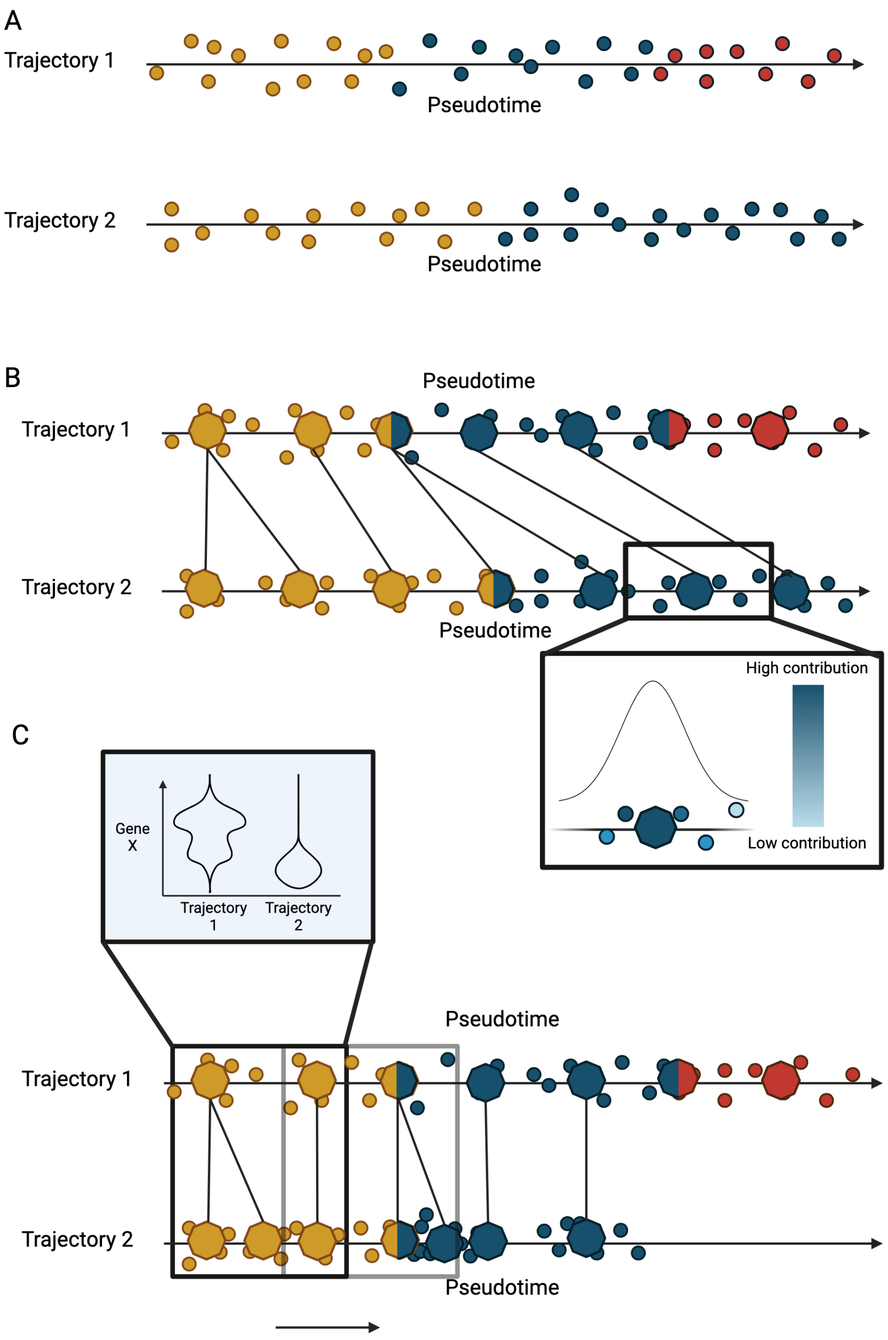

Supplementary Figure 1: Graphical overview of the TrAGEDy process.
Trajectory Inference is carried out on two datasets that share a common process but have a difference in condition (A). TrAGEDy samples gene expression across interpolated points of the trajectory, with cells with closer pseudotime values to the interpolated points contributing more to their gene expression (B). TrAGEDy then aligns the pseudotime of the interpolated points then the cells, finally performing a sliding window comparison between cells at similar points in aligned pseudotime, thereby extracting DE genes (C).

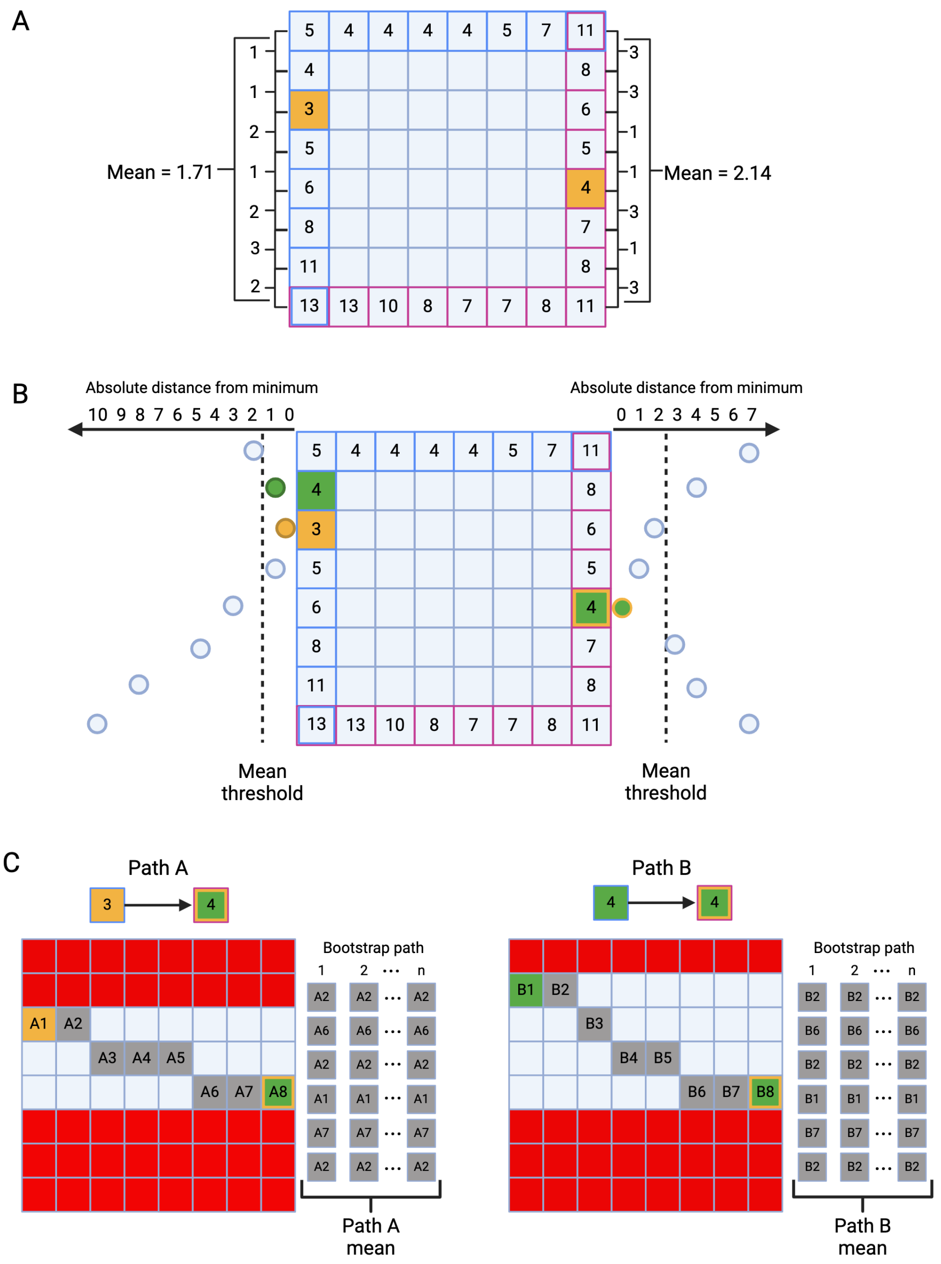

Supplementary Figure 2: Graphical explanation of the TrAGEDy process of identifying the optimal start and end points.

TrAGEDy identifies the optimal start and end points by first finding the slice of the score matrix from the start (outlined in blue) and end (outlined in purple) which contains the lowest dissimilarity score match (box coloured orange). TrAGEDy then finds the mean/medians of the score differences between adjacent matches on the start and end slice (A). The means/medians are then used as threshold for considering which matches are the optimal start and end points, with matches whose dissimilarity score is less than the threshold and before the current start match and after the current match (green box) are considered as possible start/end matches (B). For each of the possible paths through the data TrAGEDy performs the following steps. First, it removes any matches (shown in red) that fall outwith the select start and end points and then performs Dynamic Time Warping (DTW) on the cut score matrix. The matches included in the DTW path are then bootstrapped and the mean of the bootstrapped paths is calculated (C).

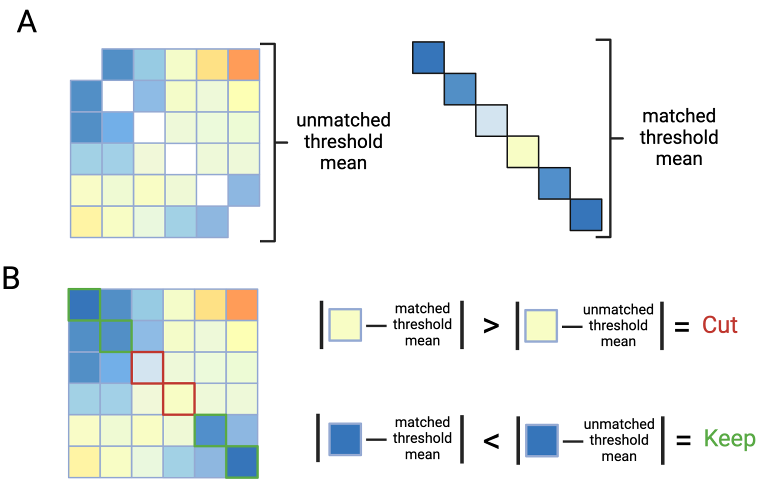

Supplementary Figure 3: Graphical explanation of the TrAGEDy process of cutting highly dissimilar matches.

To remove matches from the path which are not optimal TrAGEDy goes through the following process. An unmatched threshold and matched threshold are created by taking the mean/median of scores of the unmatched and matched points respectively (A). Simply, if the absolute difference between a matches score is closer to the unmatched threshold than the matched threshold it is cut and if the opposite is true, it is kept (B).

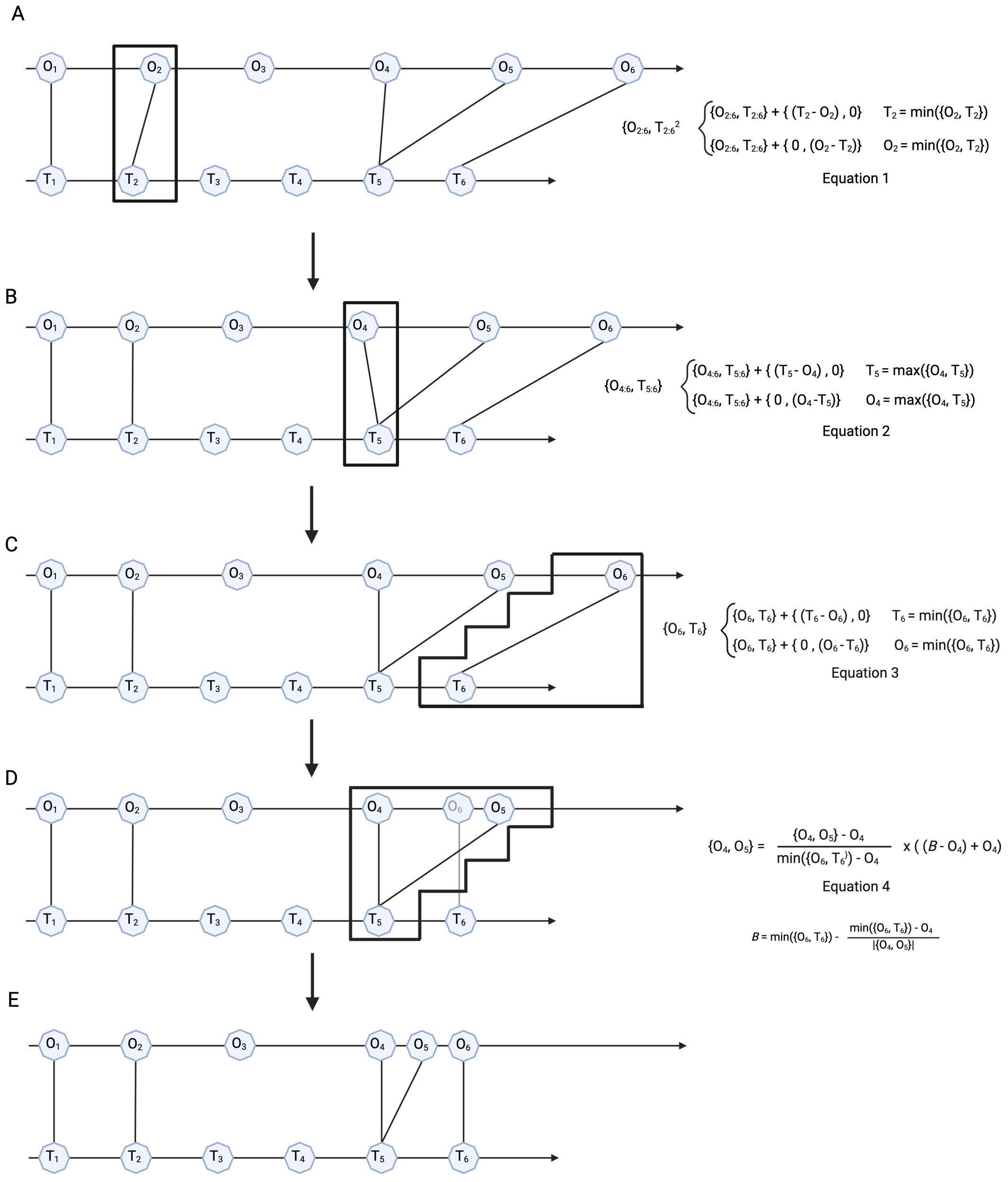

Supplementary Figure 4: Graphical explanation and example of the alignment of interpolated point pseudotime values, from the DTW calculated path.

There are two types of movement that can occur when aligning pseudotime values, an individual match (A, B & C) or a multi-match (D). For the individual match, the pseudotime value of the interpolated point with the highest value (and all the subsequent interpolated points) are brought down by the difference between the lower pseudotime value and the higher pseudotime value. For multi-match points, the match that occurs after it is adjusted then the first match in the multi-match. All the interpolated points that are matched to the same interpolated point are then scaled between pseudotime of the initial match in the multi-match and the pseudotime of the next match modified by the difference between the two, normalised by the number of interpolated points to be scaled. This then gives us aligned pseudotime values for all the interpolated points (E).

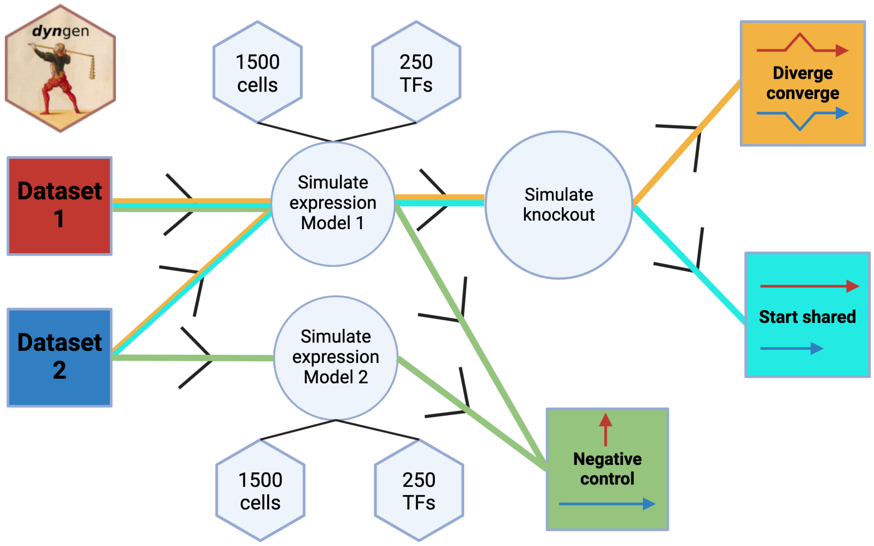

Supplementary Figure 5: Pipeline describing the Dyngen simulation strategy
Schematic showing the process of generating the three different topologies of alignment datasets, each with 1,500 cells and 250 transcription factors which drive the simulated process. For the diverge converge and start shared datasets, two datasets were generated using the same simulated expression model before knockouts were simulated for these datasets. For the negative control dataset, the expression of the two datasets was simulated using two different models (A).

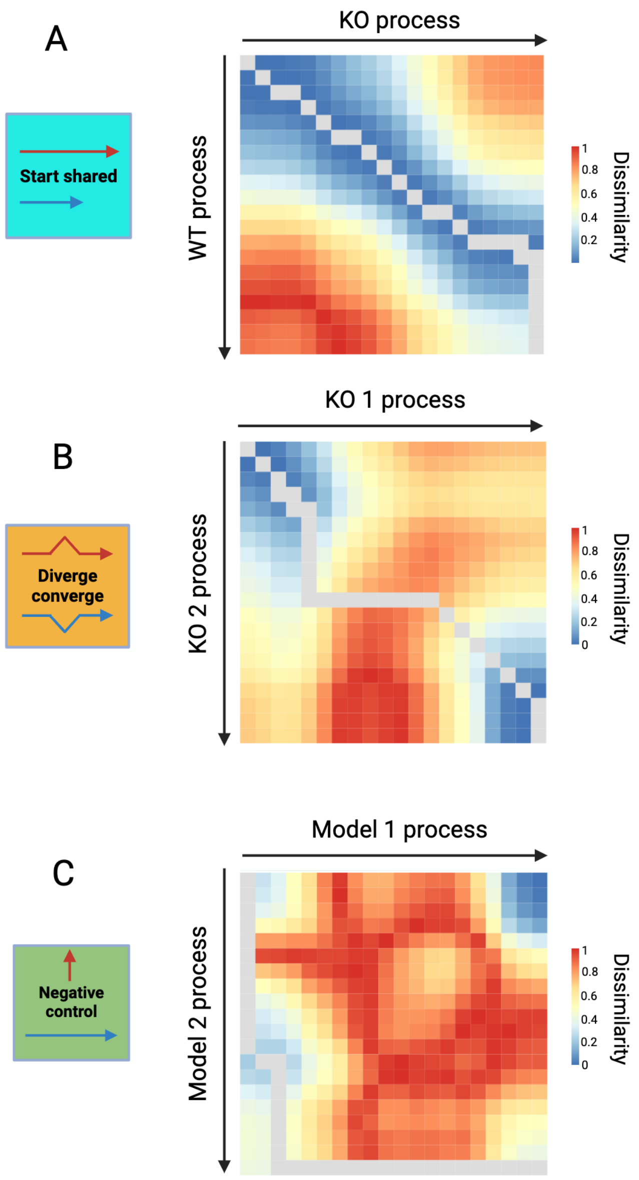

Supplementary Figure 6: cellAlign alignments of simulated datasets using Pearson correlation dissimilarity score. CellAlign alignments of the three Dyngen simulated start shared (A), diverge converge (B) and negative control datasets (C) when Pearson correlation is used as the cost function. Each box on the heatmap represents the transcriptional dissimilarity score of an interpolated point of each of the two datasets. The grey line represents the path of optimal alignment.

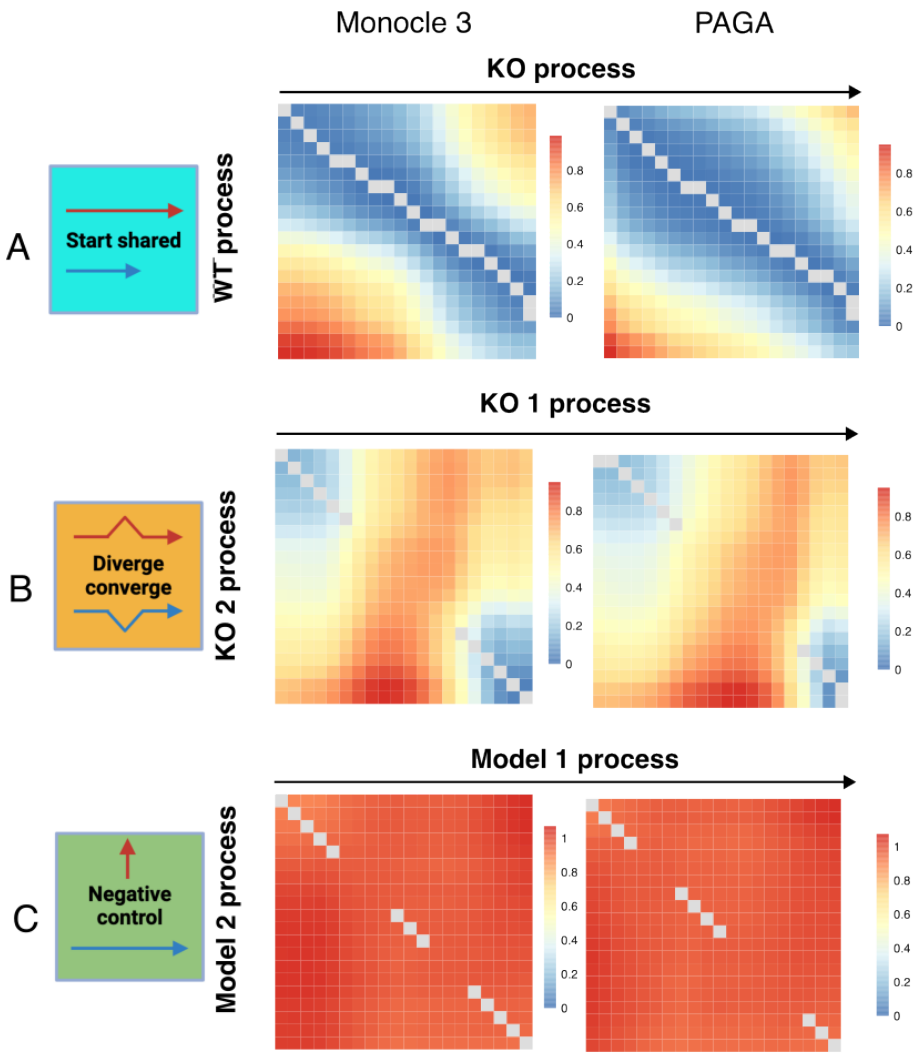

Supplementary Figure 7: TrAGEDy alignments of simulated datasets using Monocle 3 and PAGA derived pseudotime

TrAGEDy alignments of the three Dyngen simulated start shared (A), diverge converge (B) and negative control datasets (C) using Monocle 3 (column one) and PAGA (column two) derived pseudotime. Each box on the heatmap represents the transcriptional dissimilarity score (as assessed by Spearman's correlation) of an interpolated point of each of the two datasets. The grey line represents the path of optimal alignment.

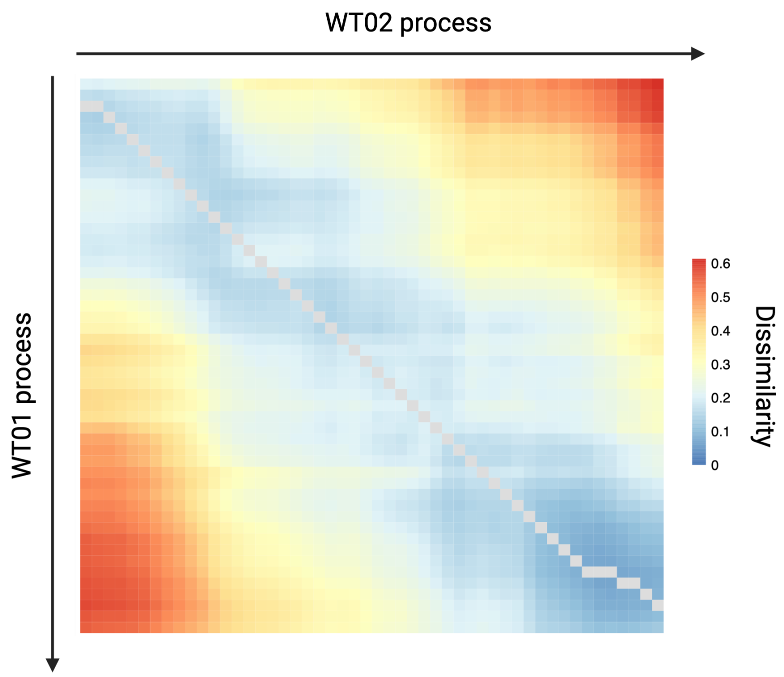

Supplementary Figure 8: TrAGEDy alignment of biological replicates of WT slender to stumpy *Trypanosoma brucei* transition.

TrAGEDy alignment of the WT01 and WT02 slender to stumpy *Trypanosoma brucei* development trajectories. Dissimilarity in gene expression of interpolated points was calculated using Spearman correlation with blue meaning low dissimilarity and red meaning high dissimilarity.

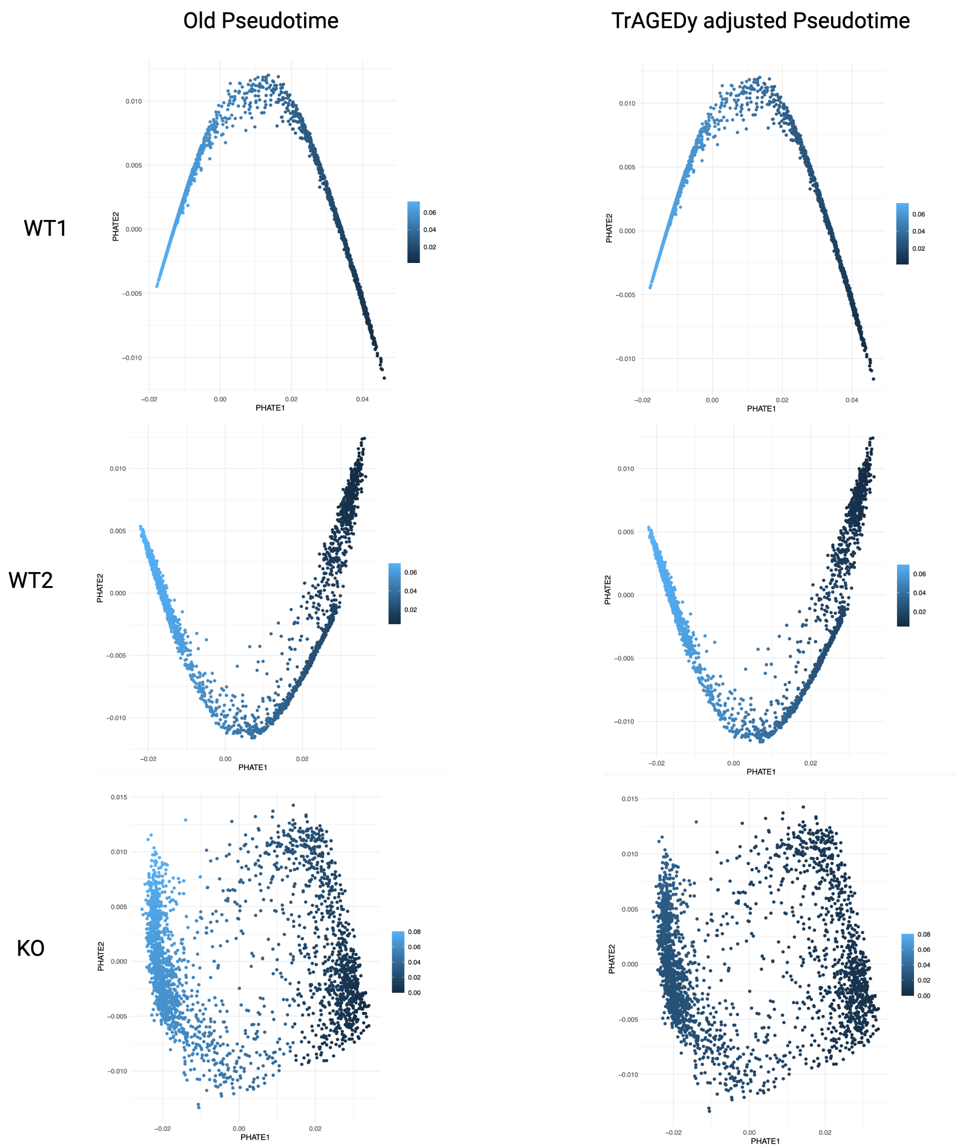

Supplementary Figure 9: Pre and post-TrAGEDy pseudotime values across PHATE space for the WT and *ZC3H20* KO *T. brucei* datasets

PHATE reductions of the individual sequencing runs of the WT (WT1 and WT2) and *ZC3H20* KO (KO) *T. brucei* scRNA-seq datasets, where each point is a cell coloured by its pseudotime value, pre (left column) or post-TrAGEDy (right column).

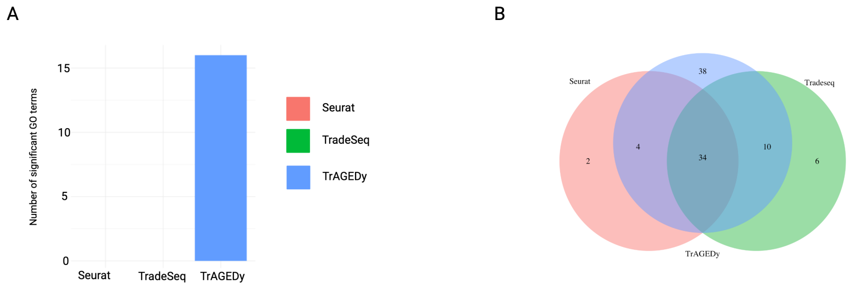

Supplementary Figure 10: Further GO term analysis of WT vs *ZC3H20* KO *Trypanosoma brucei* bloodstream form development.

Barplot showing the number of significant Gene Ontology (GO) terms (Benjamini-Hochberg adjusted p-value < 0.01) returned when GO enrichment analysis was carried out using clusterprofiler on the unique DE genes returned by TrAGEDy, TradeSeq and Seurat (A). Venn diagram showing the intersections of significant GO terms (Benjamini-Hochberg adjusted p-value < 0.01) found by the three methods using clusterprofiler on all the DE genes returned by TrAGEDy, TradeSeq and Seurat (B).

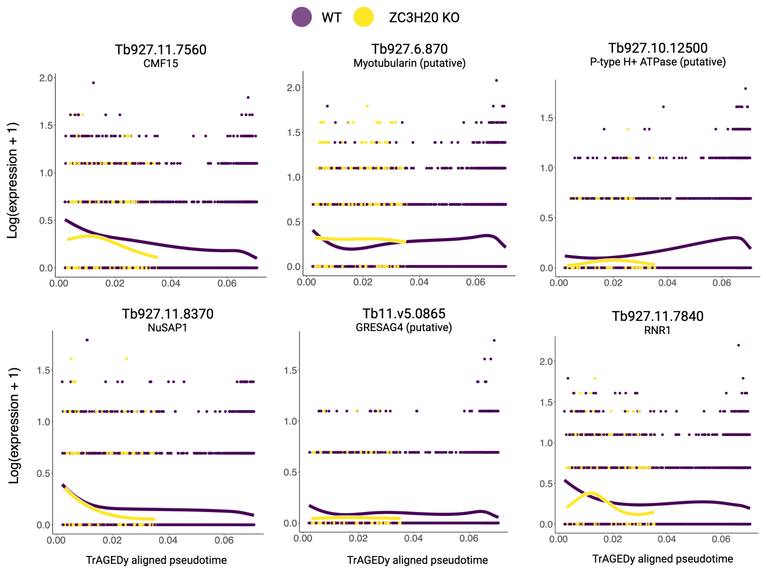

Supplementary Figure 11: Modeled smoothed expression of selected TrAGEDy DE genes across the TrAGEDy aligned pseudotime axis.

TradeSeq plotSmoother plots showing the expression changes that occur across the WT (yellow) and *ZC3H20* KO (purple) trajectories using the TrAGEDy adjusted pseudotime axis. The 6 genes plotted were only identified as being significantly DE by TrAGEDy and are also plotted in Fig.**??**E.

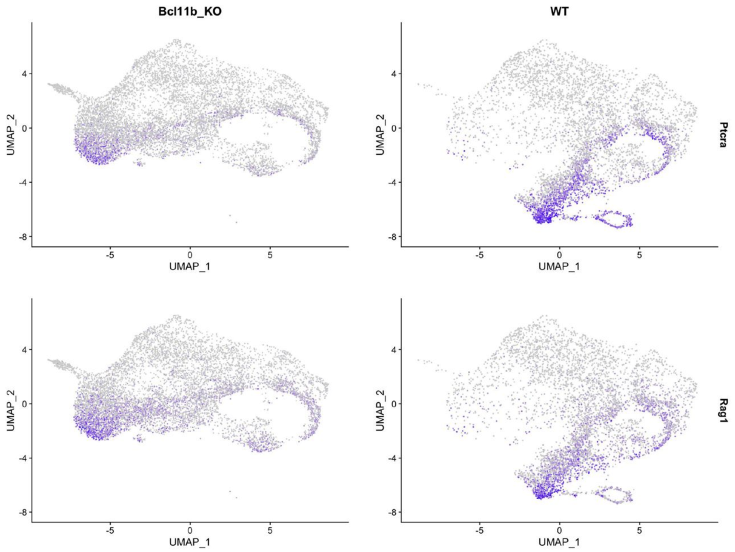

Supplementary Figure 12: Expression of markers associated with DN3 stage of T cell development in *Bcl11b* KO and WT T cells.

UMAP showing the normalised gene expression levels of *Ptcra* (pre-T cell receptor α chain) and *Rag1* (recombination activating gene 1) in T cells under WT (right column) and *Bcl11b* KO (left column) conditions.

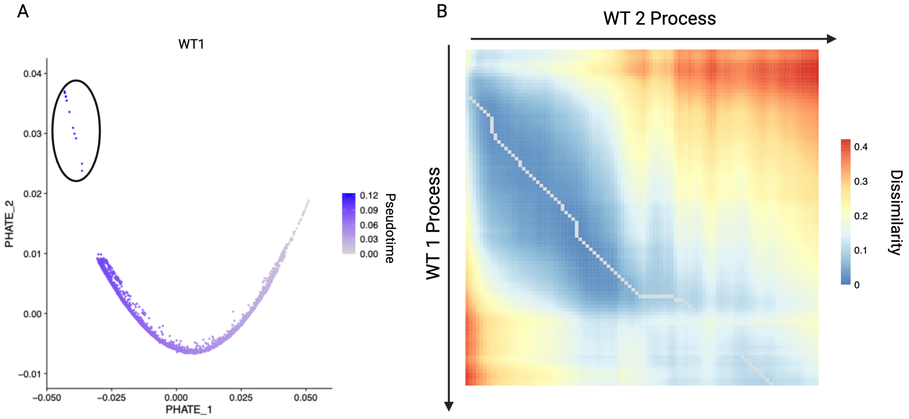

Supplementary Figure 13: Impact of outlier cells on TrAGEDy alignments.

Plot showing the PHATE embeddings of the WT1 T cell dataset with each cell coloured by its cell type annotation. Circle identifies cells which are separate from the main body of the trajectory (A). TrAGEDy alignment of the WT1 and WT2 T cell datasets when the cells circled in A are kept in the trajectory. Dissimilarity in gene expression of interpolated points was calculated using Spearman correlation with blue meaning low dissimilarity and red meaning high dissimilarity.

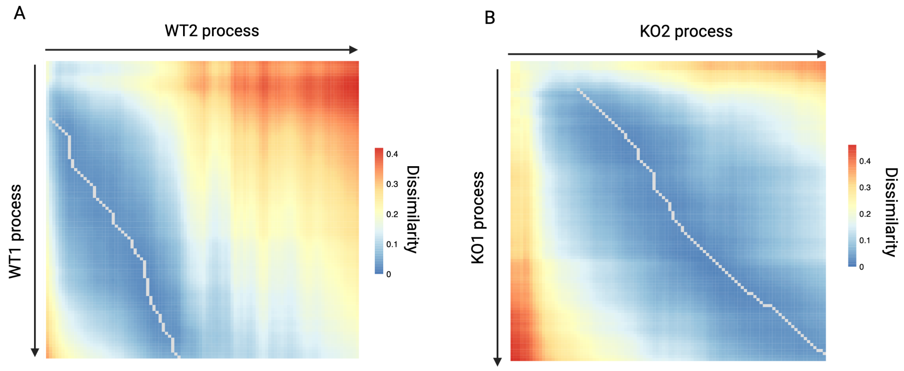

Supplementary Figure 14: TrAGEDy alignment of WT and *Bcl11b* KO T cell sequencing run trajectories.

TrAGEDy alignment of the WT1 and WT2 T cell development trajectories (A) and the *Bcl11b* KO1 and *Bcl11b* KO2 T cell development trajectories (B). Dissimilarity in gene expression of interpolated points was calculated using Spearman correlation with blue meaning low dissimilarity and red meaning high dissimilarity.

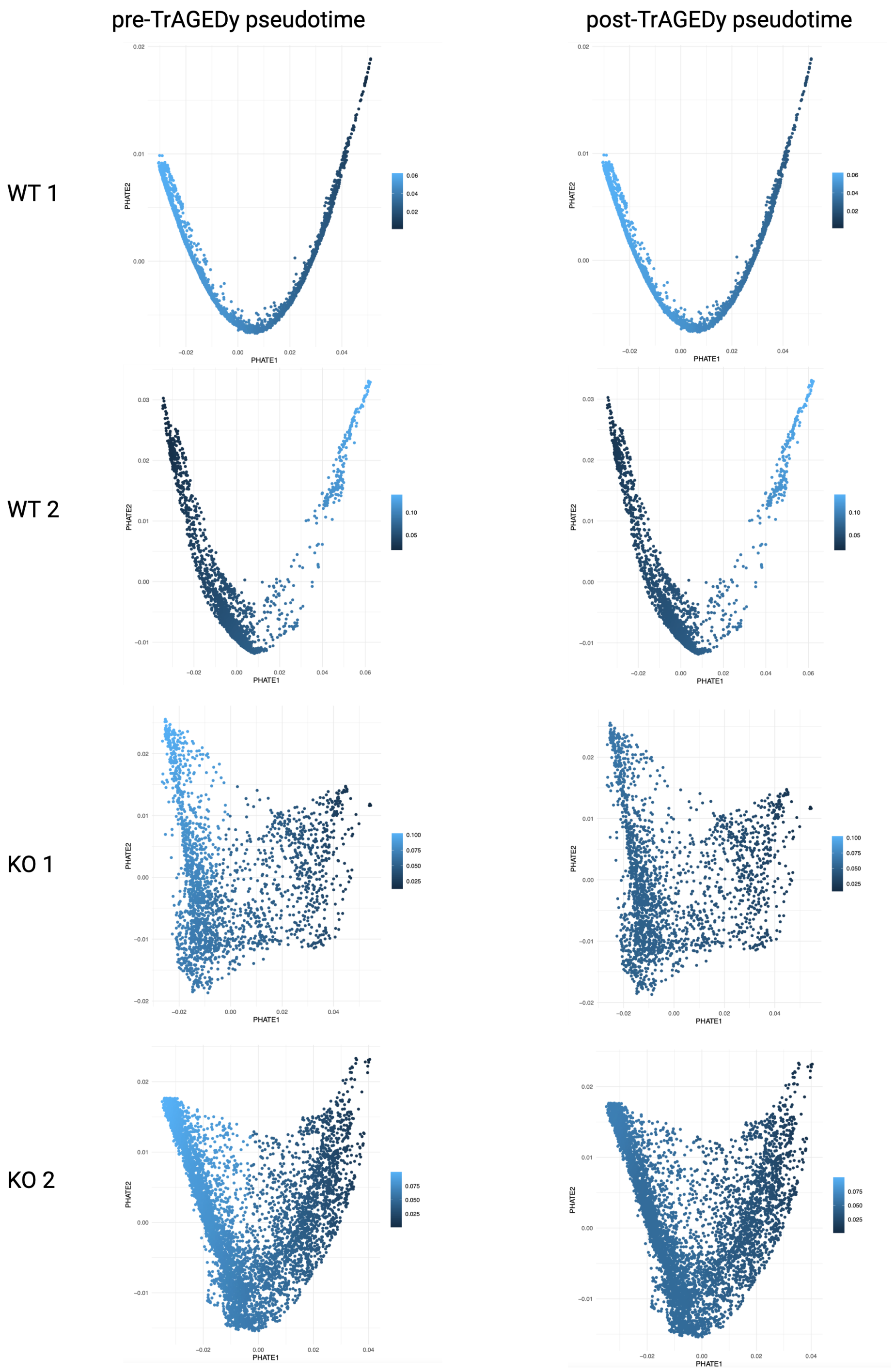

Supplementary Figure 15: Pre and post-TrAGEDy pseudotime values across PHATE space for WT and *Bcl11b* KO T cell datasets

PHATE reductions of the individual sequencing runs of the WT (WT1 and WT2) and *Bcl11b* KO (KO1 and KO2) T cell scRNA-seq datasets, where each point is a cell coloured by its pseudotime value, pre (left column) or post-TrAGEDy (right column).

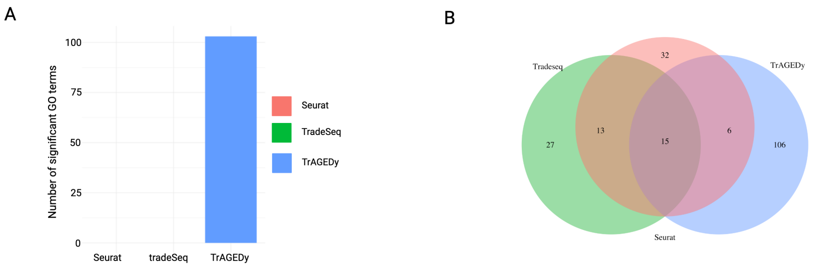

Supplementary Figure 16: Further GO term analysis of WT vs *Bcl11b* KO T cell development.

Barplot showing the number of significant Gene Ontology (GO) terms (Benjamini-Hochberg adjusted p-value < 0.01) returned when GO enrichment analysis was carried out using clusterprofiler on the unique DE genes returned by TrAGEDy, TradeSeq and Seurat (A). Venn diagram showing the intersections of significant GO terms (Benjamini-Hochberg adjusted p-value < 0.01) found by the three methods using clusterprofiler on all the DE genes returned by TrAGEDy, TradeSeq and Seurat (B).

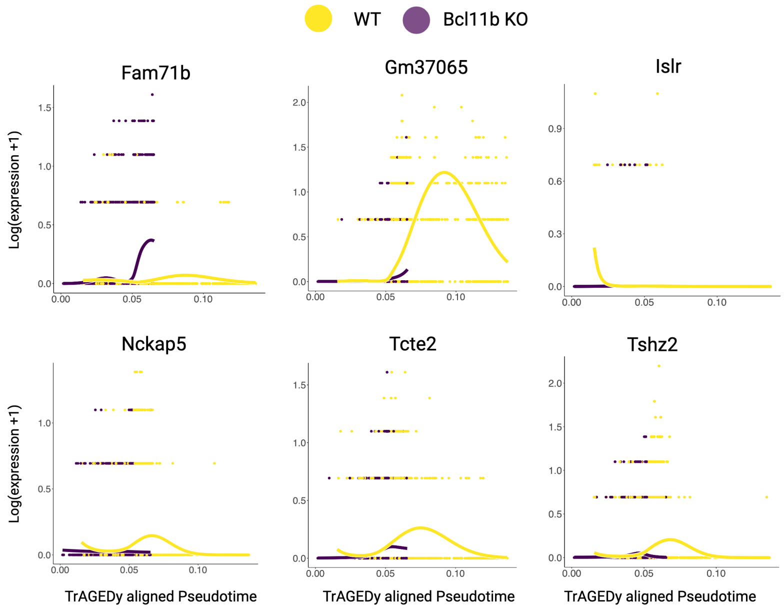

Supplementary Figure 17: Modeled smoothed expression of selected TrAGEDy DE genes across the TrAGEDy aligned pseudotime axis.

TradeSeq plotSmoother plots showing the expression changes that occur across the WT (yellow) and *Bcl11b* KO (purple) trajectories using the TrAGEDy adjusted pseudotime axis. The six genes plotted were only identified as being significantly DE by TrAGEDy and are also plotted in Fig.??E.

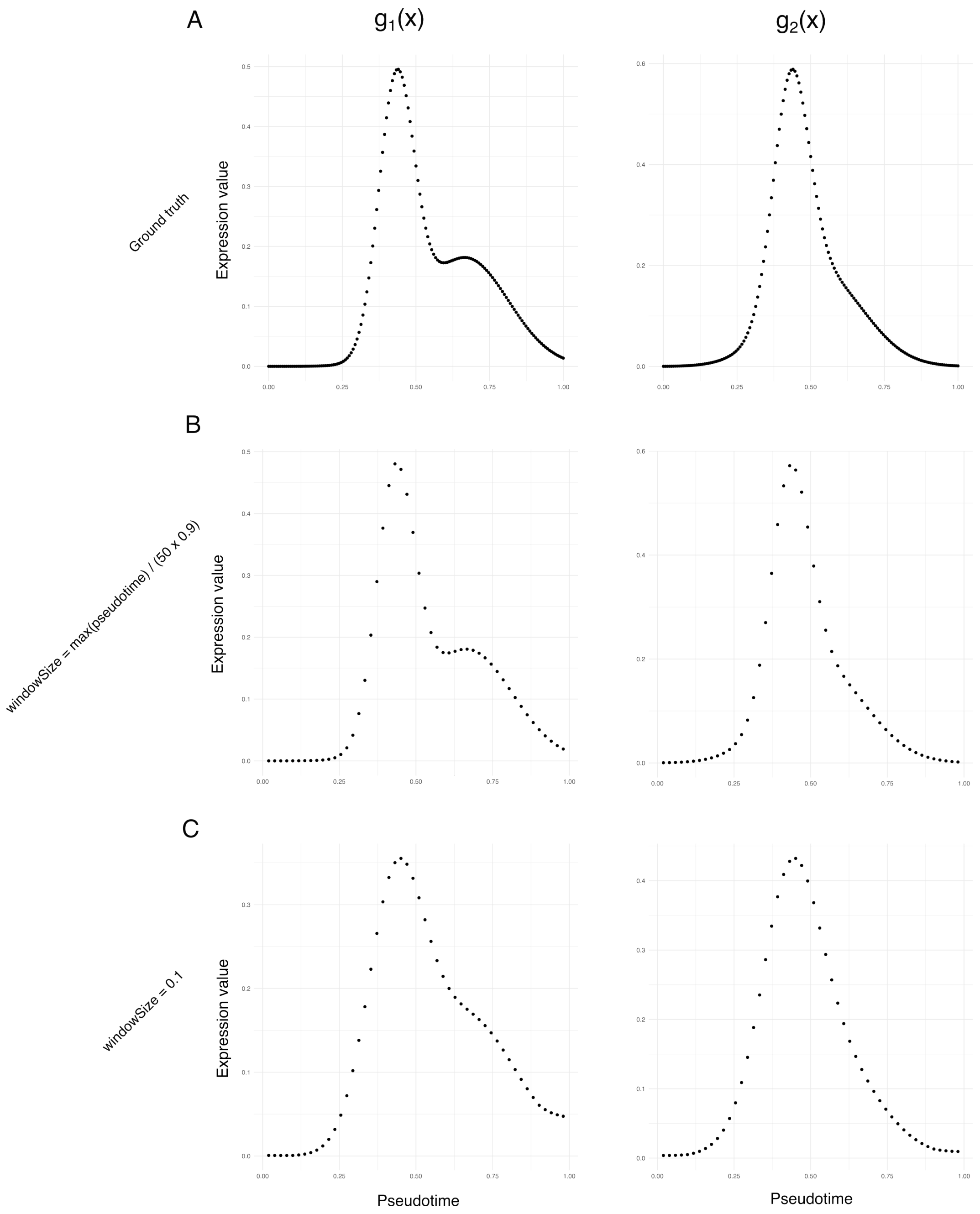

Supplementary Figure 18: Interpolated smoothed expression across different window sizes

Expression values of two functions across pseudotime (scaled between 0 and 1) (A) and the smoothed expression values using cellAligns method of creating interpolated points where the window size is equal to the pseudotime maximum divided by the number of interpolated points multiplied by 0.9 (B) and the window size is equal to 0.1 (C).

4 Optimising hyperparameters

4.1 Window size

The choice of window size depends on whether the user is interested in local or global alignment of the data. If the user is interested in global changes, then a larger window size may give the resolution required.

We find that cellAligns recommendations for a window size equal to 10% of the pseudotime axis (i.e. 0.1 on a pseudotime axis interval of [0,1]) is not enough to get accurate resolution on the gene expression changes, so we recommend a smaller window size. In our manuscript we take the window size to be:

$$windowSize = \frac{max(cellPseudotime)}{nInterpolatedPoints \times 0.9}$$

As an example of where a larger window size can fail, we constructed two functions:

$$g_1(x) = f(x) + j(x)$$

$$g_2(x) = f(x) + h(x)$$

where:

$\mu_1 = 1.5$
$\mu_2 = 5$
$\mu_3 = 3$
$\sigma_1 = 0.9$
$\sigma_2 = 2.2$

$$f(x) = \frac{1}{\sigma_1\sqrt{2\pi}}e^{-0.5(\frac{(x-\mu_1)}{\sigma_1})^2}$$

$$j(x) = \frac{1}{\sigma_2\sqrt{2\pi}}e^{-0.5(\frac{(x-\mu_2)}{\sigma_2})^2}$$

$$h(x) = \frac{1}{\sigma_2\sqrt{2\pi}}e^{-0.5(\frac{(x-\mu_3)}{\sigma_2})^2}$$

When plotted, $g_1(x)$ has two noticeable peaks, while $g_2(x)$ only has one (Supplementary Fig.18A). When the interpolated point smoothed expression is plotted with a window size of 0.1, this peak is smoothed out of the profile (Supplementary Fig.18C). If a window size recommended by TrAGEDy is used, this peak is noticeable (Supplementary Fig.18B).

4.2 Number of interpolated points

The number of interpolated points given to the trajectories should be based on how complex the biological process being aligned is i.e. the more complex and long the process, the more interpolated points that should be assigned. We recommend around 10-20 interpolated points per cluster in the dataset as a good initial setting.

5 Biological interpretation of *T. brucei* results

Furthermore, the genes captured only by TrAGEDy that were more highly expressed in the WT were consistent with the slender to stumpy transition. These included the Arginine Kinase *AK3* (Tb927.9.6210) (Ooi et al. 2015), RNA binding protein *RBP38* (Tb927.11.5850) (Sbicego et al. 2003), and delta-1-pyrroline-5-carboxylate dehydrogenase (*DP5CDH*) (Tb927.10.3210) (Supplementary file 1).

TrAGEDy identifies many known key processes which are required for *T. brucei* survival in the tsetse fly. While bloodstream forms do not wholly rely on glycolysis for its energy needs, most of its energy is produced through this pathway (Durieux et al. 1991 & Taleva et al. 2023). In contrast to mammals, the tsetse fly is a low glucose environment (Qiu et al. 2018), and the stumpy form is preadapted to suit these low glucose conditions (Grinsven et al. 2009). In the tsetse fly, the stumpy derived PCFs can breakdown amino acids for energy, in particular proline (Evans and Brown 1972, Weelden et al. 2003, Lamour et al. 2005). The parasite converts proline into glutamate through the action of proline dehydrogenase (*PDH*) and *DP5CDH* with glutamate being further converted, through the action of glutamate-dehygrogenase, into 2-oxoglutarate, which can be used as an energy source in PCFs (Weelden et al. 2003, Mantilla et al. 2017). Only TrAGEDy captures *DP5CDH* as being significantly upregulated in the WT at the end of the shared process, with TrAGEDy and Seurat both capturing glutamate dehydrogenase as being

significantly upregulated in the WT towards the end of the shared process. None of the methods captured *PDH* as being DE at any point in the shared process. TrAGEDy thus paints a more complete picture of the proline catabolism pathway, which is active in the preadapted stumpy forms, than Seurat or TradeSeq.

6 Biological interpretation of T cell results

Genes with higher expression in the WT that only TrAGEDy identified as being DE include those associated with T cell signaling (*Cd28*, *Ctla4*) (Sansom 2000) and cell cycle associated genes (*Top2a*, *Dut*, *Cenpa*) (Giotti et al. 2019). The genes only TrAGEDy identified as being significantly higher expressed in *Bcl11b* KO cells include pro-apoptotic factors (*Ikbip*, *G0s2* and *Stk4*) (Hofer-Warbinek et al. 2004, Welch et al. 2009, Cinar et al. 2007) and transcription factor (*Gata3* (Supplementary file 8).

TrAGEDy uniquely captured many cell-cycle associated genes as significantly upregulated in the WT compared with *Bcl11b* KO. After β-selection, before they begin to rearrange their TCR α chain, T cells undergo a proliferative burst and then become quiescent (Kreslavsky et al. 2012, Hwang et al. 2020). In accordance with this, cell cycle genes are mostly DE and upregulated in the WT in the final two windows (windows 5 and 6). Furthermore, the GO terms that only TrAGEDy captured were mainly associated with cell cycle function. This indicates that TrAGEDy has managed to capture the transient changes in proliferative state as WT T cells pass β-selection and prepare to rearrange their TCR α chain. Furthermore, TrAGEDy uniquely detected an increase in expression of *Cd28* and *Ctla4* in the WT compared to the *Bcl11b* KO. This finding further solidifies the conclusion that the WT T cells are able to pass β-selection while the *Bcl11b* KO are unable to, as *Ctla4* expression increases after T cell activation and an increase in *Cd28* expression has been seen following successful β-selection in maturing T cells (Linsley et al. 1992 & Teague et al. 2010).
